# Distinct features of the host-parasite interactions between nonadherent and adherent *Trichomonas vaginalis* isolates

**DOI:** 10.1371/journal.pntd.0011016

**Published:** 2023-01-03

**Authors:** Hong-Ming Hsu, Yen-Yu Yang, Yu-Hsin Huang, Chien-Hsin Chu, Ting-Jui Tu, Yen-Ting Wu, Chu-Jen Chiang, Shi-Bing Yang, Daniel K. Hsu, Fu-Tong Liu, Jung-Hsiang Tai

**Affiliations:** 1 Department of Tropical Medicine and Parasitology, College of Medicine, National Taiwan University, Taipei, Taiwan; 2 Division of Infectious Diseases and Immunology, Institute of Biomedical Sciences, Academia Sinica, Taipei, Taiwan; 3 Division of Neuroscience, Institute of Biomedical Sciences, Academia Sinica, Taipei, Taiwan; 4 High School Talent Student in Life Science Project at Academia Sinica and Taipei Municipal Chenggong High School, Taipei, Taiwan; 5 Department of Dermatology, University of California Davis, Sacramento, California, United States of America; National University of Singapore, SINGAPORE

## Abstract

Cytoadherence of *Trichomonas vaginalis* to human vaginal epithelial cells (*h*VECs) was previously shown to involve surface lipoglycans and several reputed adhesins on the parasite. Herein, we report some new observations on the host-parasite interactions of adherent versus nonadherent *T*. *vaginalis* isolates to *h*VECs. The binding of the TH17 adherent isolate to *h*VECs exhibited an initial discrete phase followed by an aggregation phase inhibited by lactose. *T*. *vaginalis* infection immediately induced surface expression of galectin-1 and -3, with extracellular amounts in the spent medium initially decreasing and then increasing thereafter over the next 60 min. Extracellular galectin-1 and -3 were detected on the parasite surface but only the TH17 adherent isolate could uptake galectin-3 via the lysosomes. Only the adherent isolate could morphologically transform from the round-up flagellate with numerous transient protrusions into a flat amoeboid form on contact with the solid surface. Cytochalasin D challenge revealed that actin organization was essential to parasite morphogenesis and cytoadherence. Real-time microscopy showed that parasite exploring and anchoring on *h*VECs via the axostyle may be required for initial cytoadherence. Together, the parasite cytoskeleton behaviors may collaborate with cell surface adhesion molecules for cytoadherence. The nonadherent isolate migrated faster than the adherent isolate, with motility transiently increasing in the presence of *h*VECs. Meanwhile, differential histone acetylation was detected between the two isolates. Also, TH17 without Mycoplasma symbiosis suggests that symbiont might not determine TH17 innate cytoadherence. Our findings regarding distinctive host-parasite interactions of the isolates may provide novel insights into *T*. *vaginalis* infection.

## Author summary

The cytoadherence of *T*. *vaginalis* onto *h*VECs from urogenital tracts has not been fully elucidated, so we developed an integrated assay platform to multifacetedly monitor the distinct behaviors and host-parasite interactions of nonadherent and adherent *T*. *vaginalis* isolates. The binding assay revealed that the trophozoites from the adherent isolate discretely bound to *h*VECs in the initial infection, later aggregating into clusters on the *h*VEC monolayer, and this cluster formation was reduced by lactose. *T*. *vaginalis* infection triggered galectin-1 and -3 secretion from *h*VECs to bind to the parasite, then galectin-3 was engulfed in the lysosome inside the trophozoites of the adherent isolate. Imaging analysis revealed that cytoskeleton reorganization is important for flagellate-amoeboid transformation and consequential cytoadherence. Also, the parasite behaviors of exploring and anchoring to *h*VECs via its axostyle contribute to the initial stage of cytoadherence. The parasite may also sense environmental factors from the host cell to change migration. Moreover, differential acetylation indicates the presence of epigenetic variation between the adherent and nonadherent isolates. No mycoplasma was detected in the T1 or TH17 isolates indicating that the symbiont may be not a determining factor for TH17 intrinsic cytoadherence. These distinct features and differential host-parasite interactions may be correlated to the pathogenesis of this parasite.

## Introduction

*Trichomonas vaginalis* causes the most common sexually-transmitted disease of nonviral origin in humans [1]. The infection usually manifests mild symptoms or is asymptomatic [2], but in severe cases, it could lead to preterm delivery, abortion, low birth weight, or stillbirth [3]. Moreover, trichomoniasis has been recognized as a risk factor for the transmission of human immunodeficiency virus and papillomaviruses [4–5], as well as the development of progressive cervical and prostate cancers [6–9]. Typically, the infection is treated with metronidazole [10], which is also used to treat gram-negative bacterial infections or luminal giardiasis and amoebiasis [10]. With increasing reports of drug-resistant clinical isolates, trichomoniasis is emerging as a threat to public health [11].

*T*. *vaginalis* is an unusual parasite that only survives in humans as trophozoites, without an alternate life stage to escape from the host. Research on the host-parasite interactions often relies on cell culture systems [12]. Numerous studies on the virulence and pathogenesis of the parasite have suggested that the trophozoites may penetrate the mucosal layer and adhere to human epithelial cells in the urogenital tracts, where they could breach epithelial barriers, lyse red blood cells, and degrade complements and humoral factors to elicit chronic inflammation [12, 13]. Adverse outcomes are likely to be mediated through the contact-dependent cytotoxicity of the parasite and several secretory proteins, such as various proteases and a macrophage migration inhibition factor-like protein [14–18]. However, the cellular mechanisms underlying the parasite virulence as well as host responses have not been fully elucidated.

Cytoadherence of the trophozoites to epithelial cells in the urogenital tract is probably one of the crucial steps for the parasite to establish infection. It has been extensively studied based on evaluating the binding capacity of trophozoites from various isolates to host cells under specified conditions [18–21], with a plethora of functional proteins identified to be involved in cytoadherence, like two membrane-associated proteins (BAP1 and BAP2) [20], a cadherin-like protein [22], secreted proteases [18, 23], BspA and Pmp domain-containing proteins [21, 24], and several hypothetical surface proteins [19] were identified and proposed to contribute to the cytoadherence of this parasite. By contrast, surface lipoglycans on the parasite have been proven to be the ligand for human galectin-1 or galectin-3 [25, 26].

The morphology of *T*. *vaginalis* transforms from a round-shaped flagellate to an irregular amoeboid when it encounters a solid surface or host cells. The cell morphology is shaped by the cytoskeleton through the complicated interplay of structural proteins and effectors [27]. Decades ago, the inhibitor of actin polymerization was shown to reduce the parasite binding to the plastic surface [28], but the molecular mechanism has not been determined.

Regarding the epigenetic regulation of *T*. *vaginalis*, epigenome mapping revealed that HDAC inhibition induces genome-wide changes in acetylation at histone H3 Lys27 (H3K27Ac) and tri-methylation at histone H3 Lys4 (H3K4me3), which globally regulate gene transcriptions in *T*. *vaginalis* [29]. In the presence of iron, ChIP-qPCR exhibited H3K27Ac and H3K4me3 highly enriched within the coding region of iron-responsive *ap65-1* (TVAG_340290) and *pfo* (TVAG_198110) genes, which may involve cytoadherence [30]. Moreover, the acetylation of histone H3 (H3Ac) was enriched around the transcription start sites of BAP1 and BAP2 adhesion molecule genes in the *T*. *vaginalis* adherent B7268 strain but not in the less-adherent G3 strain [20]. When the G3 strain was treated with the histone deacetylase inhibitor, trichostatin A (TSA), there was a higher level of H3Ac and open chromatin detected around the transcription start sites of BAP1 and BAP2 genes with increasing IBP39 binding the initiators, thereby activating transcription of BAP genes. Simultaneously, TSA treatment increased parasite aggregation and cytoadherence of the less-adherent G3 strain supporting the relevance of epigenetic regulation and *T*. *vaginalis* cytoadherence. However, whether histone modifications play roles in the process of host-parasite interaction or may serve as the markers to differentiate the pathogenesis of various *T*. *vaginalis* isolates remain to be determined.

This study aimed to explore some neglected phenomena in the host-parasite interaction by fluorescence microscopy in conjunction with scanning electron microscopy and real-time imaging, observing some distinct features of cytoadherence, motility, and histone acetylation in the *T*. *vaginalis* adherent and nonadherent isolates, as well as the destination of extracellular galectins induced by parasite infection, and the new roles of the actin-centric cytoskeleton and the microtubular axostyle underlying cytoadherence. These findings further contribute to our understanding of host-parasite interactions.

## Materials and methods

### Cultures

*T*. *vaginalis* was maintained in TYI medium supplemented with 10% calf serum as previously described [31]. The parasite with an initial density of ~1×10^5^ ml^-1^ was grown overnight to the mid-logarithmic phase (1.2~1.5×10^6^ ml^-1^) at 37°C. Two *T*. *vaginalis* isolates were used in this study, including T1 (nonadherent isolate) [19, 21, 32, 33, 34] with flagellate form only freely swimming in the medium suspension, and TH17 (adherent isolate) with vigorous flagellate-amoeboid transformation mainly adhering tightly on the solid surface in culture tubes and a smaller population remained at the flagellate form (S1 and S2 Videos). The TH17 was isolated from a symptomatic vaginitis pregnant woman in 1979 (National Taiwan University Hospital) and inoculated into TYI medium containing 1,000 U penicillin, 1,000 μg/ml streptomycin, 2.5 μM amphotericin B, and 0.1% agar with daily passaging over two weeks to remove microbial contamination. Then, the trophozoite maintained in TYI medium was aliquoted and stored in liquid nitrogen. A Mycoplasma-positive strain, PM1 (an isolate from Dr. Jung-Hsiang Tai, Institute of Biomedical Sciences, Academia Sinica) was used as the positive control in PCR and fluorescence microscopy for examining Mycoplasma symbiosis. Immortalized human vaginal epithelial cells (*h*VECs, VK2/E6E7, ATCC CRL-2616) and HeLa cells were respectively maintained in semi-defined keratinocyte serum-free medium (KSFM) (GIBCO-BRL 17005–042, ThermoFisher Scientific) and DMEM high glucose medium (12100046, ThermoFisher Scientific) with 10% fetal bovine serum at 37°C and 5% CO_2_.

### The binding assay to evaluate cytoadherence

Trophozoites were labeled with 5 μM of carboxyfluorescein diacetate succinimidyl ester (CFSE, CellTrace) as described by the supplier (ThermoFisher Scientific) and washed in phosphate buffered saline (PBS) twice to remove unbound dye. *h*VECs were cultured in a 12-well plate to a ~70% confluent monolayer. *T*. *vaginalis* trophozoites re-suspended in the fresh medium were inoculated at a defined multiplicity of infection (moi) with *h*VECs. The spent medium was aspirated and unbound trophozoites were removed by gently washing each well with PBS twice at different time points. Cells were fixed with 4% formaldehyde in PBS and observed by fluorescence microscopy.

### Scanning electron microscopy (SEM)

Samples were prepared as described [35]. In brief, trophozoites were co-incubated with *h*VECs on a coverslip and fixed at intervals with 4% formaldehyde and 2.5% glutaraldehyde in 0.1 M sodium phosphate buffer overnight. After washing twice in sodium phosphate buffer, samples were sequentially dehydrated in an ethanol gradient from 30 to 100%. The samples were immersed in liquid carbon dioxide prior to critical point drying and gold coating, then observed by an environmental scanning electron microscope (FEI Quanta 200).

### Enzyme-linked immunosorbent assay (ELISA)

Galectin-1 and galectin-3 secreted from human cells during the host-parasite interaction were quantified by ELISA [36, 37]. In brief, standards and serial-diluted supernatants recovered from human cultures challenged with *T*. *vaginalis* were incubated in 96-well plates precoated with 50 μl of goat anti-human galectin-1 (1 μg/ml, R&D Systems AF1152) or rabbit anti-human galectin-3 antibody (1 μg/ml, GeneTex) at room temperature for 1 hr. The plates were washed 3× in PBS before reacting with biotinylated anti-human galectin-1 (1 μg/ml, R&D Systems, BAF1152) or anti-human galectin-3 (1 μg/ml, R&D Systems, BAF1154) antibodies at room temperature for 2 hr. The plates were washed 3× with PBST (0.05% Tween 20), then 50 μl of HRP-Avidin (1,000×, BioLegend) was added into each well and incubated at room temperature for 1 hr. After washing off the unbound avidin with PBST (0.05% Tween 20), 3,3′,5,5′-tetramethylbenzidine (TMB, Invitrogen) was added and the colorimetric change was monitored using a microplate spectrophotometer at OD_450_ (SpectraMax190, Molecular Devices).

### The immunofluorescence assay (IFA)

The IFA for formaldehyde-fixed cells was performed as previously described [38]. In brief, the primary reaction was performed using the rabbit anti-human galectin-1 (1,000×) [39], rabbit anti-human galectin-3 (1,000×, Genetex), mouse anti-α-tubulin (500×, Sigma DM 1A), rabbit anti-acetylated H3K9 (3,000×, Merck Millipore), H4H5 (2,000×, Abcam), H4K8 (4,000×, Abcam), H4K12 (3,000×, Abcam) or H4K16 (3,000×, Abcam) diluted in TBS containing 1% BSA, at 4°C overnight. The secondary antibody reaction was performed using the FITC-, Cy3- or Cy5- conjugated goat anti-mouse or rabbit IgG antibody (200×, Jackson ImmunoResearch) diluted in TBS containing 1% BSA, at 37°C for 1 hr. Nuclei were stained by DAPI in the mounting medium (Vector Laboratories) and the fluorescent signal was measured by confocal microscope (LSM700, Zeiss). Cell morphology was recorded by phase-contrast or differential interference contrast (DIC) mode.

The surface expression of galectin-1 and galectin-3 on host cells was examined by IFA using the anti-galectin-1 and anti-galectin-3 antibodies. For this, *h*VECs were cultured on coverslips, placed in a well of a 12-well plate at 37°C in a CO_2_ incubator until ~70% confluence. The coverslips were washed once with 1 ml pre-warmed PBS and coated with 10 μl of 0.36% agar. The plate was sealed with parafilm and transferred to a humidified chamber at 4°C for 10 min before the addition of 20–30 μl of primary antibodies. The plate was incubated in a humidified chamber at 4°C for 10 min followed by shifting to 13°C for 50 min. The coverslips were washed with 1 ml of pre-chilled PBS 3 times and fixed in 500 μl of 4% formaldehyde in the cold room for 20 min. The slides were washed with 1 ml PBS twice, followed by the addition of 20–30 μl of secondary antibody. The plate was then sealed with parafilm and kept in the humidified chamber at RT for 1 hr. The coverslips were washed with 1 ml PBS twice and rinsed in distilled water before air drying for microscopic observations.

To detect surface or internalized galectins in *T*. *vaginalis*, trophozoites labeled with 100 nM LysoTracker Red DND-99 probe (ThermoFisher Scientific) were incubated in fresh or spent KSFM medium collected from *h*VECs culture pre- or post-*T*. *vaginalis* infection at 4°C for 30 min.

### *In vitro* binding of galectins to the parasite

*T*. *vaginalis* at a density of 10^6^ ml^-1^ in PBS were incubated with 100 nM of FITC-conjugated recombinant galectin-1, galectin-3, or BSA at 4°C for 10 min [36, 39]. Cells were washed three times in PBS and then fixed with 4% formaldehyde in PBS. The fluorescent signal was measured by flow cytometry (LSRII SORP 17 color analyzer, Becton Dickinson) or fluorescence microscopy (LSM700, Zeiss).

### Real-time microscopy

Trophozoites labeled with CFSE were loaded onto host cells pre-cultured in a 35-mm glass-bottom dish, and the host-parasite interactions were monitored by real-time microscopy (LSM780, Zeiss) at a sampling rate of 1 frame per 30 sec over time as defined.

### Motility

To measure *T*. *vaginalis* motility, trophozoites from overnight cultures were loaded onto a glass slide and a thin coverslip was carefully placed on top of the medium. The motion was captured with an upright DIC microscope at a sampling rate of 10 frames per sec at room temperature, and the track displacement length (TDL) and velocity (V) were calculated by Imaris v.9.2.1 software (Oxford Instruments).

### Inhibitor treatment

The final concentration of 50 μM cytochalasin D (CytD) or 20 μM tubulin polymerization inhibitor II (TPI) was added to *T*. *vaginalis* culture and incubated at 37°C for 2 hr before assay.

### G-actin and F-actin biochemical fractionation

G- and F-actin were fractionated using an in vivo assay biochem kit (Cytoskeleton, Inc), with minor modifications. Briefly, ~3× 10^7^ trophozoites were incubated in lysis buffer (containing 1× Protease inhibitor cocktail and 200 μg/ml TLCK) with vigorous agitation at 4°C for 30 min. The total lysate was centrifuged at 1,000× *g* to remove cell debris, followed by ultracentrifugation at 100,000× *g* for 1 hr to separate the insoluble F-actin in pellet and the soluble G-actin in the supernatant for western blotting.

### Western blotting

Protein samples were separated using a 12% SDS-PAGE gel and blotted onto 0.45 μm polyvinylidene difluoride (PVDF) membranes for western blotting assay. The membrane was sequentially incubated with mouse anti-α-actin (10,000× Ac-40, Genetex), mouse anti-α-tubulin (10,000×, Sigma, DM1A), mouse anti-*Tv*CyP2 (3,000×) [40], and mouse anti-GAPDH (5,000×, a gift from Dr. John Alderete, Washington State University) and horseradish peroxidase (HRP)-conjugated goat anti-mouse secondary antibody (Jackson ImmunoResearch). Signals were detected by the enhanced chemiluminescence (ECL) system (ThermoFisher Scientific). The images were captured by the UVP ChemiDoc-815 system and quantified by UVP VisionWorks software (Analytikjena Company).

### Analysis of morphogenesis

Approximately **~**2× 10^7^ trophozoites were inoculated into a T25 culture flask containing 30 ml of TYI medium and incubated at 37°C for 1.5 hr or until 90% trophozoites were sedimented on the flask bottom. The parasite morphology was observed by phase-contrast microscopy and the proportion of spherical flagellates and irregular amoeboid form was measured in 600 trophozoites in 6 independent microscopic fields.

### Lactate Dehydrogenase (LDH) cytotoxicity assay

Host cells were cultivated in a 96-well microplate to a 90% confluency, and then inoculated with parasites at a moi of 1:3. After incubation at indicated time intervals, 100 μl medium was collected and centrifuged at 1,000× *g* to remove cell debris. The LDH activity in the supernatant was detected using a LDH cytotoxicity kit (BioChain). Briefly, 45 μl of Assay Mixture was incubated with 100 μl of the test sample for 30 min, then 50 μl of Stop Solution was added. The colorimetric signal was detected by a spectrophotometer at OD_490_ (SpectraMax190, Molecular Devices). For each assay, the medium supernatants from host cells treated with or without Lysis Solution were the positive and negative controls, respectively. The cytotoxicity (%) was measured as

$$\frac{OD490\;\left(test\;sample\right)-\;OD490\;(negative\;control)}{OD490\;\left(positive\;control\right)-OD490\;(negative\;control)}\;\times 100.$$

### PCR for Mycoplasma symbiont detection

The DNA fragments of *T*. *vaginalis 18S ribosomal RNA* and *Mycoplasma 16s rRNA* genes were amplified from the DNA extracted from the parasites by PCR with the degenerate primer pairs Tv18srRNA-f/Tv18srRNA-r and MGSO/GPO-1 [41], respectively. The DNA products were separated in a 1% agarose gel and visualized with SYBR Safe DNA Gel Stain (ThermoFisher Scientific) using the UV transilluminator (UVP Gel solo, Analytikjena Company).

Tv18srRNA-f: 5′-AAGTCTGGTGCCAGCAGCCG-3′
Tv18srRNA-r: 5′-CCCGTGTTGAGTCAAATTAAGC-3′
MGSO: 5′-TGCACCATCTGTCACTCTGTTAACCTC-5′
GPO-1: 5′-ACTCCTACGGGAGGCAGCCGTA-3′

### Statistical analysis

The relative differences in data collected between conditional samples were statistically analyzed by Student’s t-tests, unless otherwise specified. A P-value < 0.05 was considered statistically significant.

## Results

**Fig 1:**
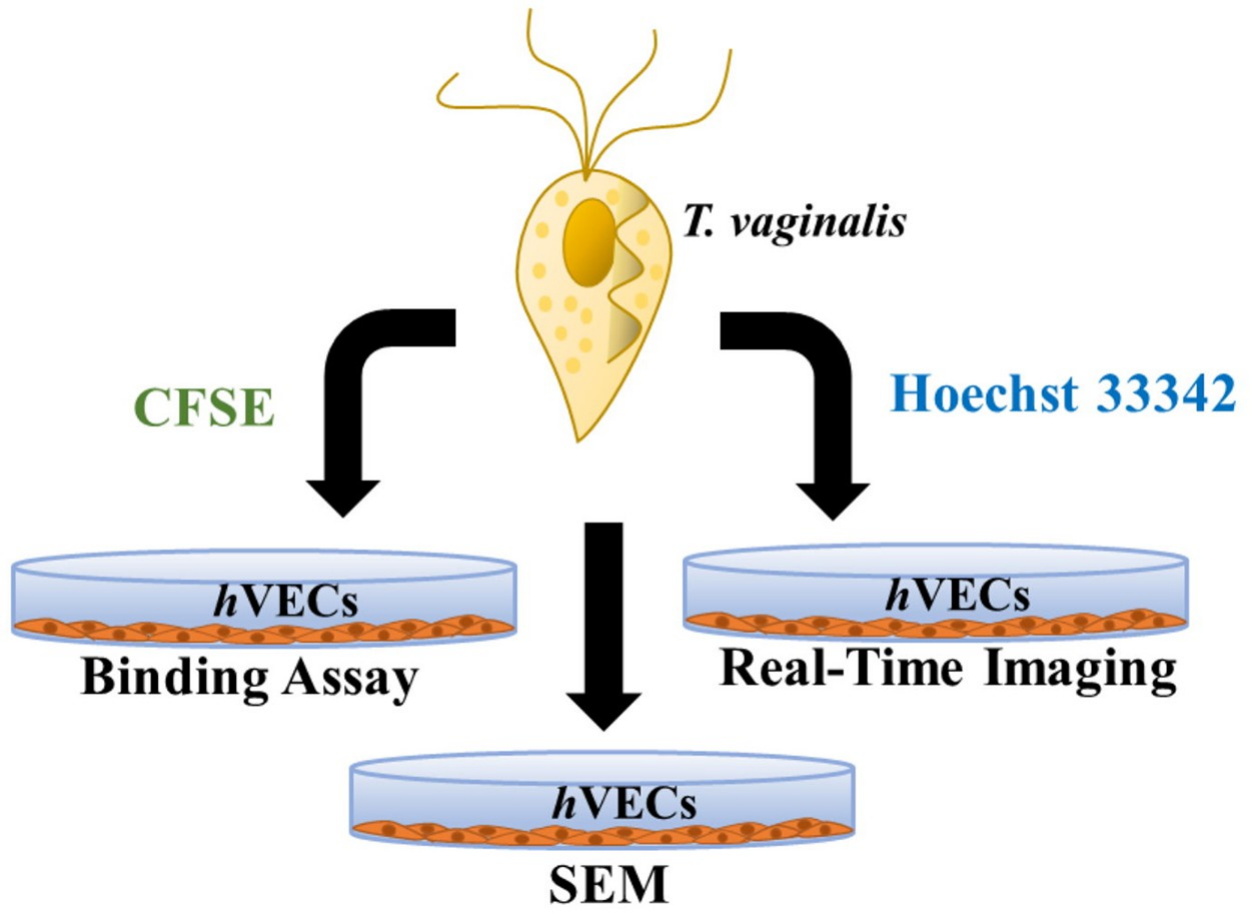
A platform to study the interactions between *T*. *vaginalis* trophozoites and human epithelial cells. A semi-quantitative binding assay in conjunction with real-time imaging and scanning microscopy (SEM) were applied to study the interactions between *T*. *vaginalis* and human urogenital tract epithelial cells. For the binding assay or real-time microscopy, trophozoites were labeled with CFSE or Hoechst 33342, respectively, while non-labeled trophozoites were explored for SEM. At defined intervals, unbound trophozoites were removed and the adherent trophozoites were fixed for microscopic examination.

This study aimed to explore the host-parasite interaction by fluorescence microscopy for the assays of conventional cytoadherence and specified proteins’ subcellular localization, by SEM to show the morphology, and by real-time imaging to observe parasite behaviors (Fig 1). Two *T*. *vaginalis* isolates were used in this study, T1 is a nonadherent isolate with only flagellate form freely swimming in the medium suspension. TH17 is an adherent isolate with vigorous flagellate-amoeboid transformation activity mainly adhering tightly on the solid surface in culture tubes with locomotion by amoeboid migration. Only a tiny TH17 population remained at the flagellate form and freely swam in the culture medium (S1 and S2 Videos).

### The kinetics of *T*. *vaginalis* cytoadherence

**Fig 2:**
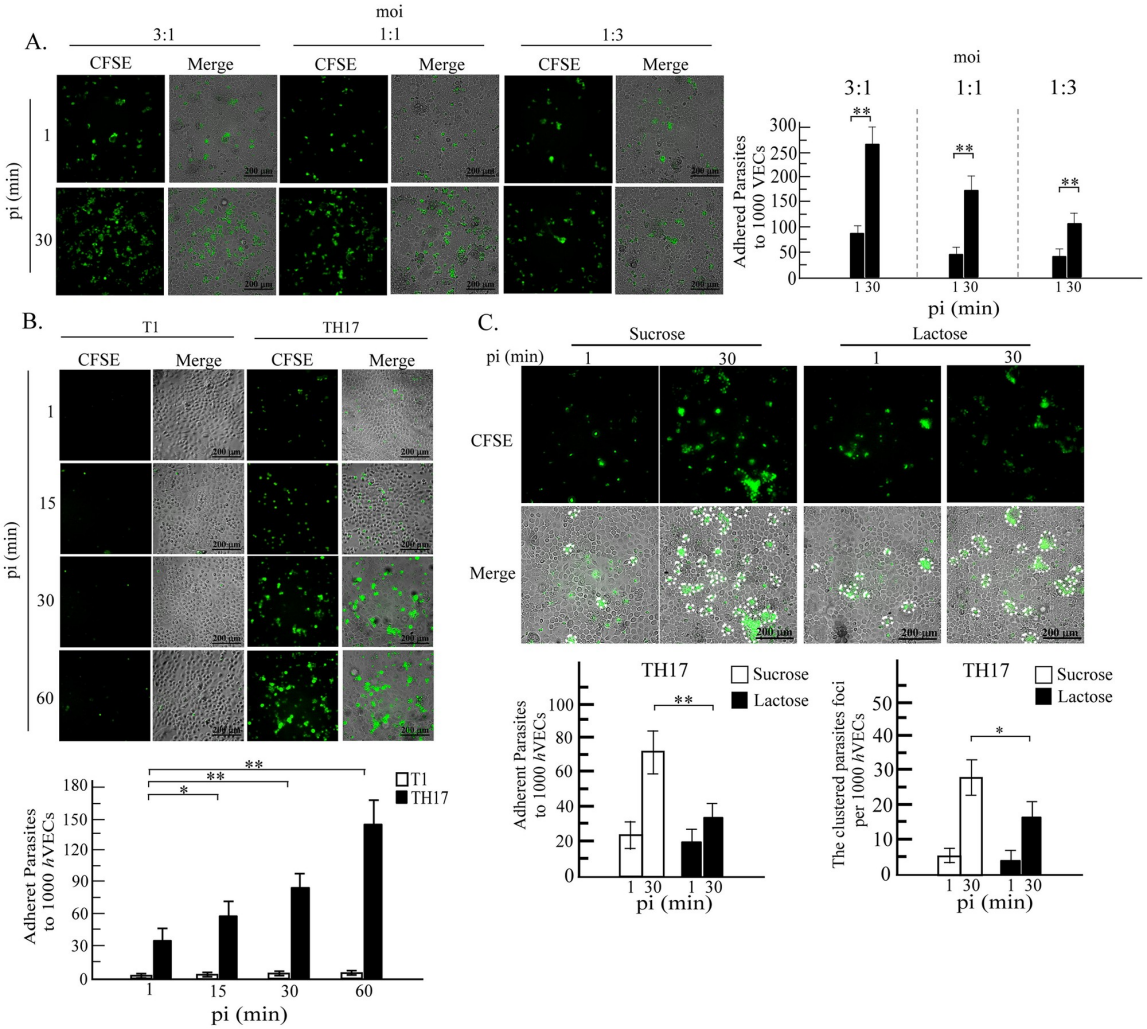
The kinetics of *T*. *vaginalis* trophozoites binding to *h*VECs. The trophozoites were labeled with CFSE and co-cultured with *h*VECs. A. trophozoites at a moi of 3 to 1, 1 to 1 or 1 to 3 were incubated with *h*VECs for 30 min. B. T1 or TH17 trophozoite were co-incubated with *h*VECs (moi 1:3) for 1, 15, 30 or 60 min. C. trophozoites were co-incubated with *h*VECs (moi 1:3) in the presence of 250 mM sucrose or lactose for 30 min and after the removal of unbound trophozoites, cell cultures were fixed for imaging by fluorescence and phase-contrast microscopy. PI, post-infection. The clustered foci with over three trophozoites are circled by white-dashed lines. The experiments were performed in triplicate and the average number of binding trophozoite or clustered foci per 1,000 *h*VECs were measured as shown in the bar graphs (mean ± SD). The statistical difference was analyzed by the Student’s t-test with P<0.05 (*), P< 0.01(**), and ns, not significant.

To evaluate cytoadherence of trophozoites from adherent versus nonadherent *T*. *vaginalis* isolates, cells were labeled with CFSE before the infection. Initially, the effect of moi for the input trophozoites on the cytoadherence of the parasite to *h*VECs was studied at 30 min post-infection (Fig 2A), showing that at a moi of 3:1, 220~300 trophozoites (equivalent to 7~10% of input) adhered per 1000 *h*VECs for the TH17 isolate. The adherent trophozoites formed clusters with 50~65 clusters per 1000 *h*VECs. At a moi of 1:1, there were 150~250 adherent trophozoites (15~25% input) per 1000 *h*VECs, with 45~55 clusters. When the moi was lowered to 1:3, 75~125 adherent trophozoites (25~40% input) per 1000 *h*VECs were observed, with 30~40 smaller clusters. The formation of the trophozoite cluster is similar to the swarming of trophozoites from the B7RC2 strain exposed to excessive recombinant galectin-1 [25]. Under the same conditions, only ~10 adherent trophozoites (less than 0.5% of input) per 1000 *h*VECs were detected for the T1 isolate, with no parasite clusters observed.

The moi at 1:3 was adopted to study cytoadherence and aggregation of trophozoites over 60 min (Fig 2B), showing ~40 and ~60 adherent TH17 trophozoites per 1000 *h*VECs at 1 and 15 min post-infection, respectively. During the early phase of infection, randomly distributed single trophozoites with a few tiny clusters were observed. At 30 and 60 min post-infection, ~80 and ~150 adherent trophozoites per 1000 *h*VECs were detected. Over time, the trophozoites were gradually observed to aggregate into 25~35 clusters at 30 min and 30~45 clusters at 60 min. These observations suggest that the infection may be divided into an initial discrete phase followed by a swarming phase.

Galectins binding *T*. *vaginalis* surface lipoglycans were previously reported as one of potential factors triggering parasite swarming [25], so the typical ligand lactose was applied to compete with the interaction between galectin and the parasite ligand [42]. In the presence of 250 mM lactose, the cytoadherence of TH17 trophozoites to *h*VECs at 1 min post-infection only varied slightly but the total number of bound trophozoites, as well as the aggregated trophozoite clusters on *h*VECs, were significantly reduced at 30 min post-infection, indicating that lactose could compete with parasite agglutination on *h*VECs (Fig 2C). Cytoadherence of the nonadherent T1 trophozoites to *h*VECs was only ~0.2 to 0.5% (Fig 2B) of the input level without an apparent swarming phenomenon under our test conditions, and this value varied slightly during the 60-min infection course. These observations suggest that *T*. *vaginalis* cytoadherence could be divided into an early galectin-independent phase and a second swarming phase, the latter of which may be dependent on secreted galectins.

### Expression and secretion of galectins during the host-parasite interaction

**Fig 3:**
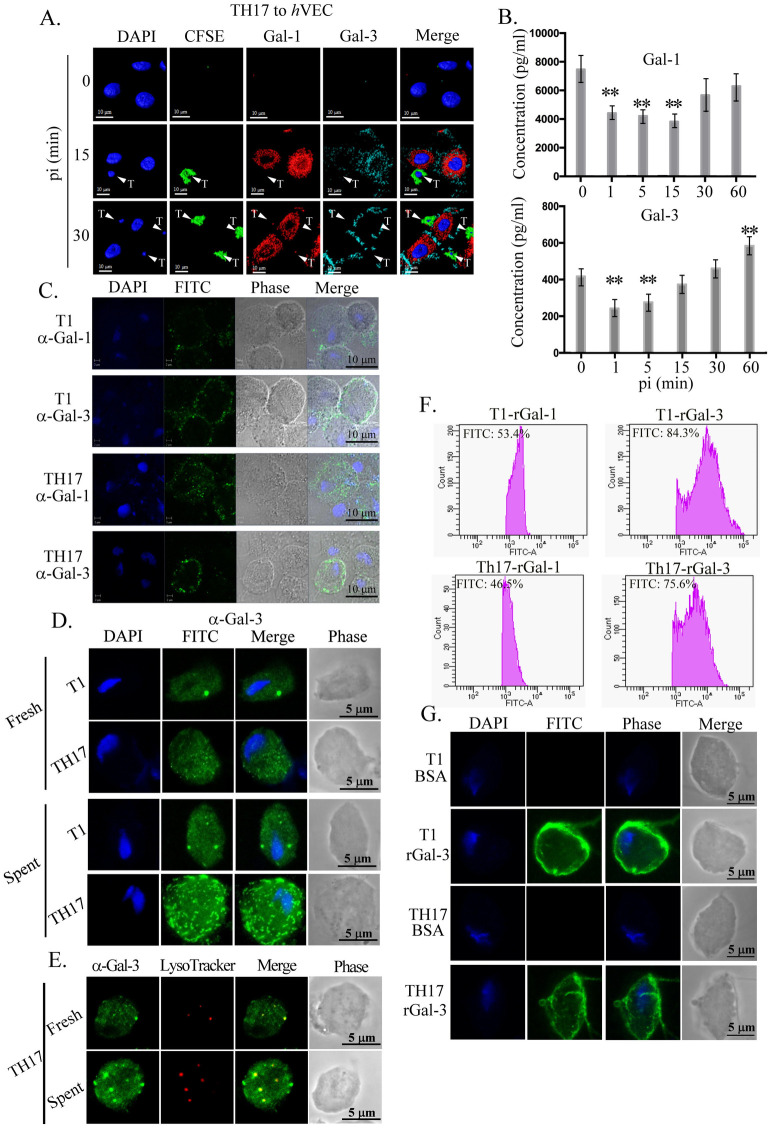
Involvement of galectins in the host-parasite interaction. A and B. Surface expression or secretion of galectins. CFSE-labeled TH17 trophozoites (green) were co-incubated with *h*VECs (moi 1:3) were sampled at intervals for the detection of galectin-1 (red) and galectin-3 (cyan) by IFA (A), while these galectins in the conditioned medium were detected by ELISA (B). PI, post-infection. Adherent trophozoites in A are indicated by white triangles. C, D, and E. T1 or TH17 trophozoites were incubated in fresh or conditioned KSFM (D) from *h*VECs cultures for 30 min. Cells were fixed for detection of galectin-1 or -3 by IFA without permeation for (C) or with permeation for (D and E) before immune detection. E. IFA co-staining with anti-galectin-3 antibody and LysoTracker. F and G. T1 or TH17 trophozoites were incubated with FITC-conjugated recombinant galectin-1 or galectin-3 and analyzed by flow cytometry (F) or fluorescence microscopy (G). The bar graph is shown by mean ± SD. All assays were repeated three times and the representative data are shown here. Differences were statistically analyzed by Student’s t-test, with *P*<0.01(**) and *P*<0.05(*).

To study whether galectin-1 and galectin-3 are involved in the early stage of host-parasite interactions, *h*VECs were infected with TH17 trophozoites and the expression of each galectin was detected on the cell surface using a live cell staining protocol. Galectin-1 and galectin-3 were localized to the apical and basal surfaces respectively on *h*VECs at 15-min and 30-min post-infection, (Fig 3A), indicating that those surface galectins are induced by the parasite infection.

To examine whether *T*. *vaginalis* infection induces the secretion of galectins from *h*VECs, the concentration of secreted galectin-1 and galectin-3 in the supernatant from human cell cultures infected with TH17 trophozoites was quantified by ELISA (Fig 3B). There was approximately ~8 ng ml^-1^ of galectin-1 in the conditioned medium from *h*VECs before the infection, which decreased by 40% at 1 min post-infection, presumably via binding to the parasite (Fig 3B upper panel), then increased up to ~80% of the original level at 30- and 60 min post-infection. By contrast, only ~0.4 ng ml^-1^ of galectin-3 was detected in the conditioned medium of *h*VECs before infection (Fig 3B lower panel). The concentration of secreted galectin-3 decreased 40% at 1 min post-infection but gradually increased up to 20% over the origin level at the subsequent time points (Fig 3B lower panel).

To test whether secreted galectins bind to the parasite, trophozoites incubated in conditioned medium from *h*VECs culture post *T*. *vaginalis* infection were analyzed by IFA (Fig 3C). For fixed cells without prior permeation, galectin-1 and galectin-3 were detected as punctate signals on the surface of T1 and TH17 trophozoites, indicating that secreted galectins may bind to *T*. *vaginalis*. With membrane permeation, only galectin-3 was significantly detected as punctate signals in the cytoplasm of TH17 (Fig 3D). As shown by real-time microscopy, galectin-3 was enriched in certain intracellular vesicles in the TH17 trophozoites (S3 Video). Co-staining with LysoTracker dye showed that the intracellular galectin-3 signal partially colocalized with the acidic lysosome-like vesicles in the TH17 adherent isolate (Fig 3E).

To see the galectin binding capacity in individuals, trophozoites were incubated with 100 nM of the FITC-conjugated recombinant galectin-1 or galectin-3 at 4°C for 10 min for flow cytometry analysis (Fig 3F). The intensity of galectin-1 measured on thousands of trophozoites varied over a ~10-fold range, whereas that of galectin-3 varied over a 100-fold range, in an isolate-independent manner. Similar results were observed by fluorescence microscopy (Fig 3G), suggesting that the abundance of galactosides on individual trophozoite surface might not be significantly different between nonadherent and adherent isolates. Whether the parasites with more galectins on their cell surfaces could more effectively bind host cells remain to be evaluated.

These observations suggest that *T*. *vaginalis* may induce the surface expression and secretion of galectins from *h*VECs. The extracellular galectins initially bind the parasite at a similar level similar in the adherent and nonadherent isolates but thereafter, only the TH17 adherent isolate can internalize galectin-3 into the cytoplasmic lysosomes. Apart from trophozoite aggregating and binding host cells [25, 26], the significance of galectins in the host-parasite interactions remains to be studied.

### Morphological changes of the parasite upon the host-parasite interactions

**Fig 4:**
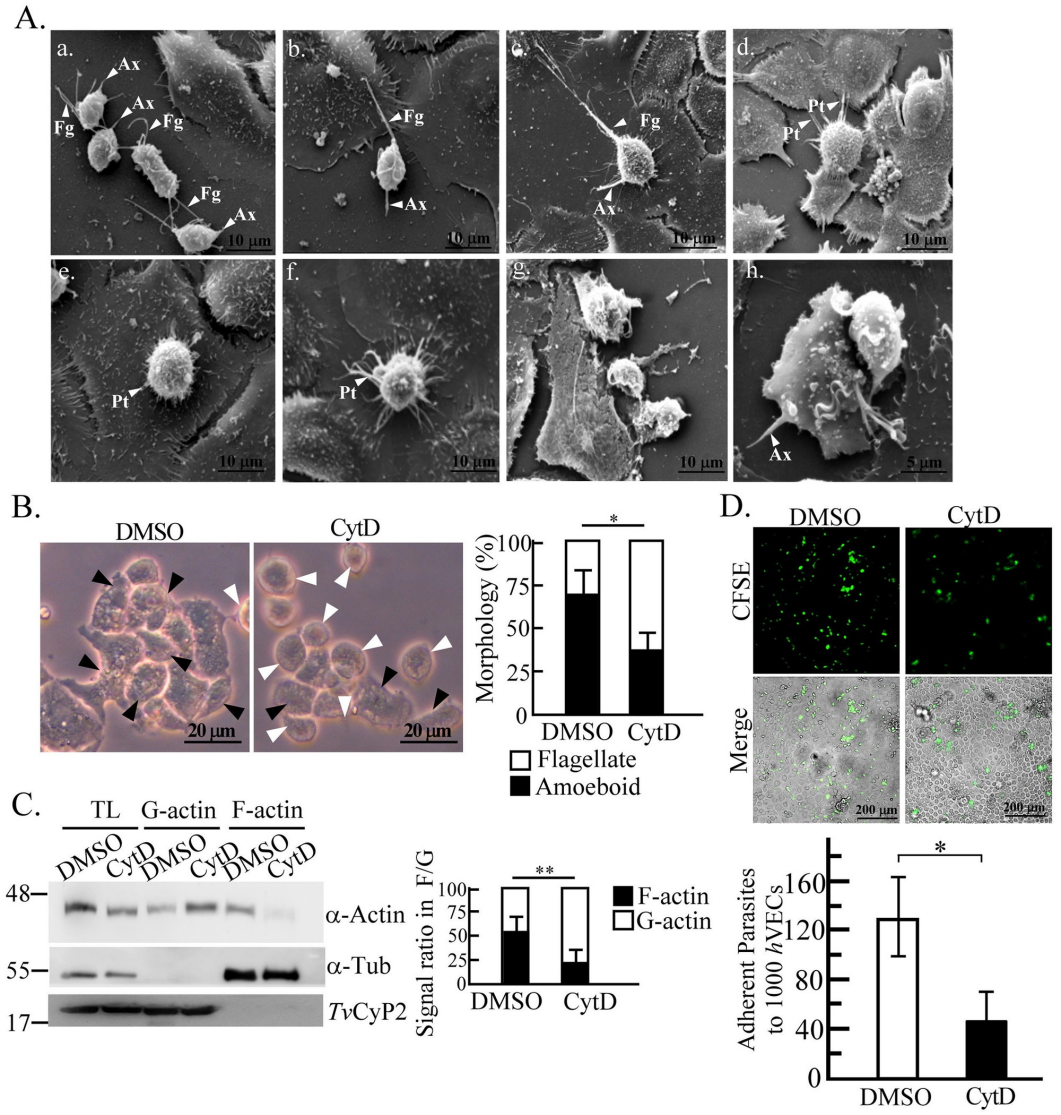
Morphological transformation of *T*. *vaginalis* on exposure to *h*VECs. A. TH17 trophozoites (a) were co-incubated with *h*VECs for 15 (b. and c.), 30 (d, e, f), or 60 min (g and h) for SEM. White triangles point to the flagellum (Fg), axostyle (Ax), or membrane protrusions (Pt). The representative morphology was observed from at least ~50 trophozoites at stages as specified in the infection course. B. The trophozoites treated with DMSO or CytD were cultured in T25 flasks for 1 hr and the morphology was monitored by phase-contrast microscopy. The black and white arrowheads respectively indicate the amoeboid and flagellate forms of *T*. *vaginalis*. The percentage of flagellate against amoeboid trophozoites was measured in 600 trophozoites within 6 microscopic fields as shown in the bar graph. C. TH17 trophozoites treated with DMSO or CytD were fractionated into F-actin and G-actin-containing fractions for western blotting. The ratio of the α-actin signal in G-actin versus F-actin was quantified as shown in the bar graph. α-Tubulin (α-Tub) and *Tv*CyP2 were detected as the purity markers of F-actin and G-actin fractions, respectively. D. The trophozoites pretreated with DMSO or CytD were co-cultured with *h*VECs (moi 1:3) for 1 hr in cytoadherence binding assay. The number of trophozoites bound per 1000 *h*VECs was measured as shown in the bar graph (mean ± SD). All experiments were repeated three times. Significant differences were statistically analyzed by the Student’s t-tests, with *P*<0.01(**) and *P*<0.05(*).

To visualize the morphology of *T*. *vaginalis* post encountering host cells, *h*VECs cultured on glass coverslips were infected with the TH17 trophozoites for SEM. After infection, individual flagellate trophozoites on the void surface, clustered flagellates contacting one another via flagella, or flagellates with elongated flagella contacting neighboring *h*VECs were observed (Fig 4Aa–4Ac). At 15 min post-infection and thereafter, the rounded-up form anchored on *h*VECs via the axostyle was observed (Fig 4Ad–4Af). Interestingly, such trophozoites displayed numerous membrane protrusions contacting one or a few adjacent *h*VECs. The amoeboid form lying flat on *h*VECs was observed at 60 min post-infection (Fig 4Agh). A small number of disintegrated *h*VECs and trophozoites were also observed. However, the activity of morphological transformation was not observed in the trophozoites of T1 nonadherent isolate, speculating that the morphogenesis may have a role in host cell binding.

When *T*. *vaginalis* was cultured in a T25 flask for 1 hr, 70% of the input TH17 trophozoites transformed from the free-swimming flagellates into the amoeboid form adhering to the surface (Fig 4B). Such a scenario was rarely seen in T1 isolate. By contrast, the amoeboid transformation was suppressed to ~35% in the TH17 trophozoites pretreated with 50 μM cytochalasin D (CytD), an actin polymerization inhibitor (Fig 4B). In the parasites treated with CytD, the F-actin polymerization was repressed by over half (Fig 4C), with the cytoadherence activity reduced to one-third of that in DMSO control cells (Fig 4D). This suggests that actin polymerization and consequent morphogenesis are involved in the capacity of this parasite adhering to *h*VECs.

### The dynamics of the host-parasite interaction

**Fig 5:**
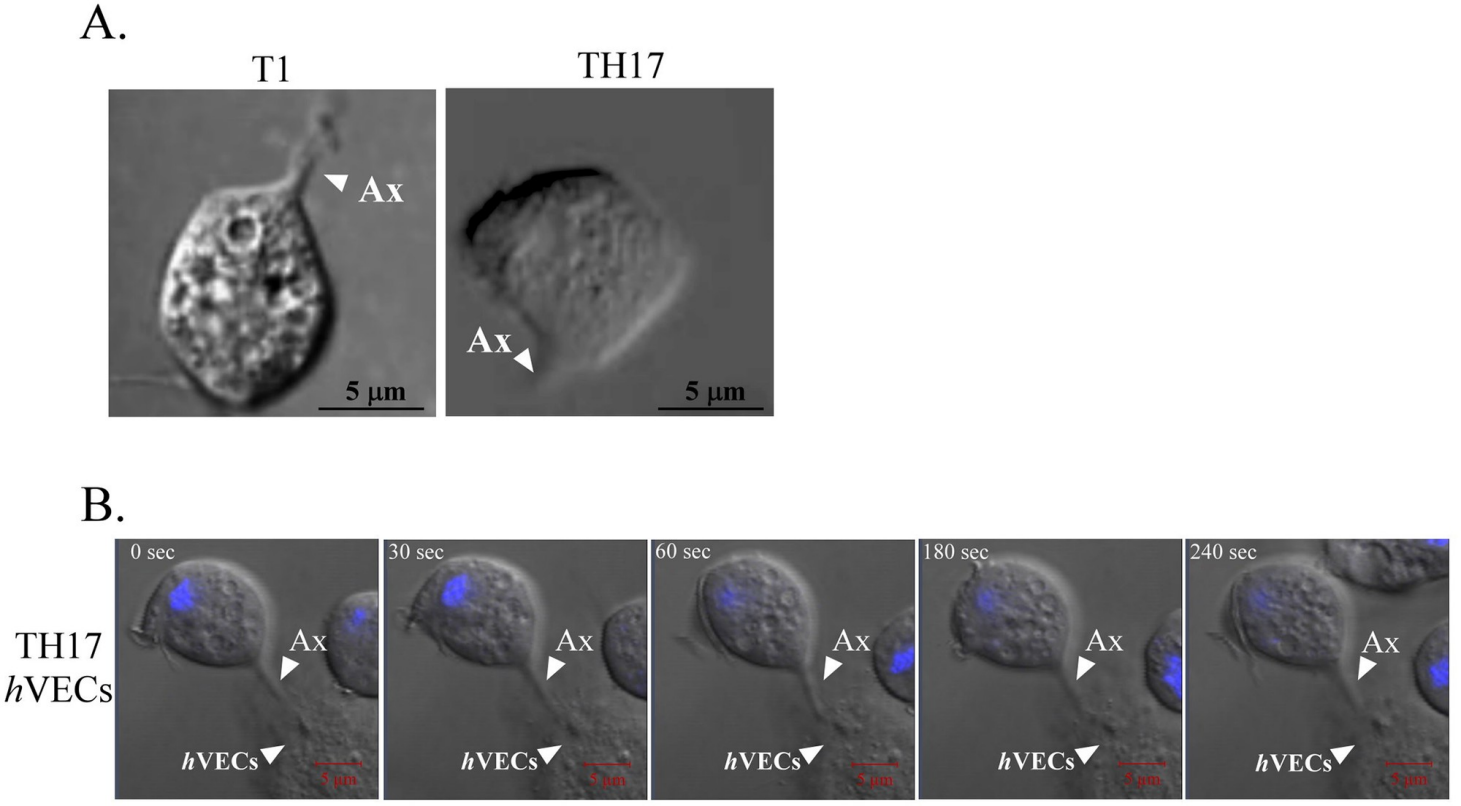
Real-time imaging of the host-parasite interactions. The behavior of T1 or TH17 trophozoites in the presence (A) or absence (B) of *h*VECs was recorded by real time-imaging (S4, S5 and S6 Videos). A representative touchdown scenario of a T1 or TH17 trophozoite on the glass surface via the axostyle is captured as shown in (A). Anchor of a Hoechst 33342-labeled TH17 trophozoite on a *h*VECs over 4 min with contiguous bulging and shrinking of its axostyle is shown in (B). Notably, the duration of the anchor varied in the individuals. Real-time images were captured by DIC at the rate of 1 frame per 30 sec over 30 min. White triangles indicate where the parasite axostyle (Ax) or human cells (*h*VECs) are located.

The dynamics of the host-parasite interaction were subsequently monitored by real-time microscopy. T1 trophozoites rapidly swung flagella and swam in the medium suspension with occasional contact to the slide surface by axostyle (Fig 5A and S4 Video). TH17 trophozoites alone zigzagged and swerved more or less in a circle, with the axostyle skipping periodically to touch the glass surface (Fig 5A and S5 Video). Upon encountering *h*VECs, TH17 trophozoites anchored on host cells via the axostyle, which were contiguously bulging and shrinking the structure, while also interacting with passing trophozoites via their flagella (S6 Video). Before transforming into the amoeboid form over time, the flagellates were randomly anchored on host cells by the axostyle (Fig 5B). By contrast, T1 trophozoites were rolling in place and floating above *h*VECs with less anchoring (S7 Video). Together, these observations suggest that adherent trophozoites can use their axostyles to explore solid surface until they encounter host cells for a lasting anchor, whereas nonadherent trophozoites are infrequently exploring environments.

### The axostyle is involved in the cytoadherence of *T*. *vaginalis*

**Fig 6:**
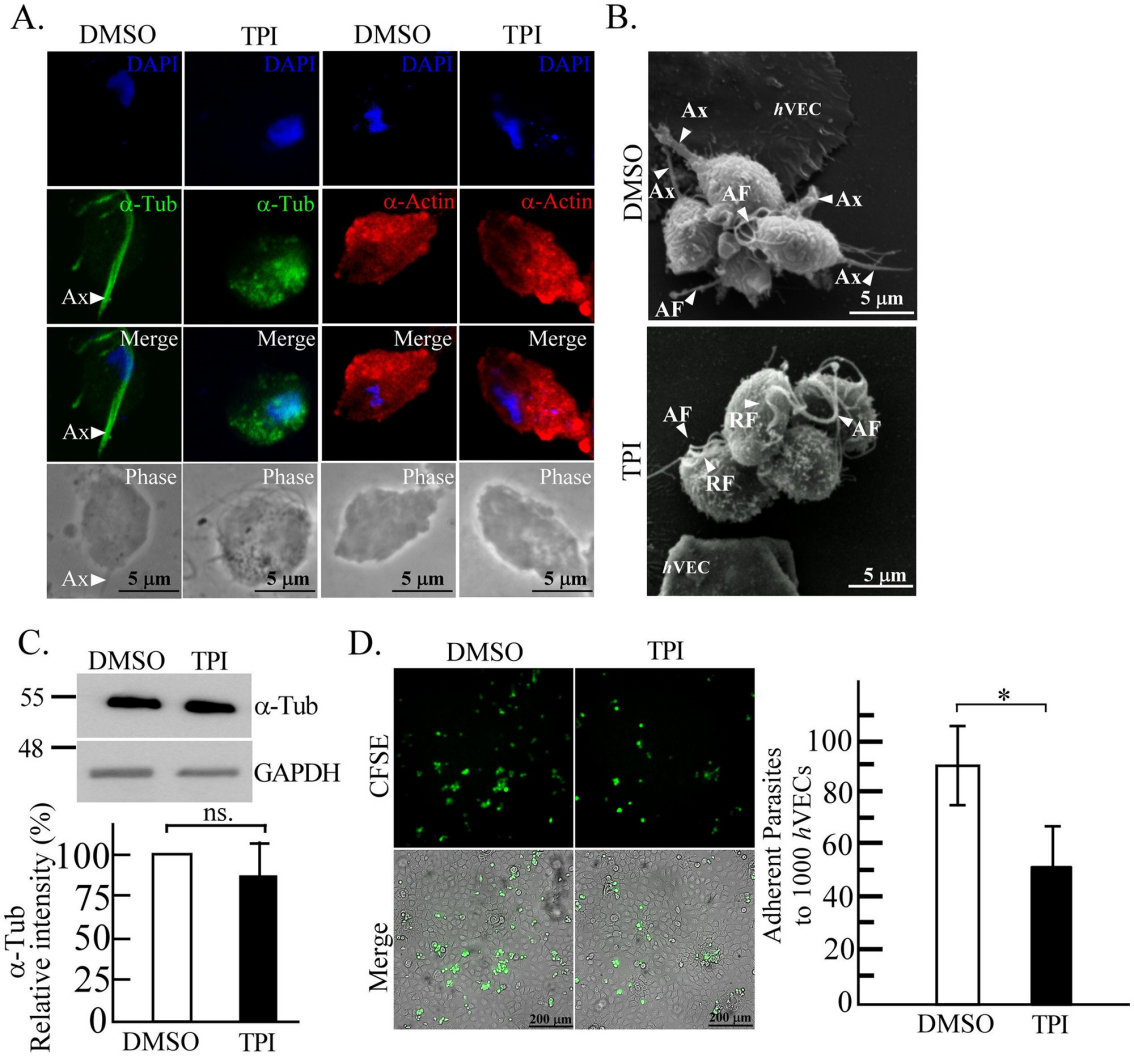
The involvement of the axostyle in the cytoadherence of the TH17 isolate. The trophozoites of TH17 isolate were pretreated with DMSO or TPI. A. the trophozoites cultured on a glass slide were examined by IFA using anti-α-tubulin or anti-α-actin antibodies. B. Axostyles in the trophozoites co-cultured with *h*VECs were observed by SEM. The arrowheads indicate axostyle (Ax); anterior flagellum (AF); recurrent flagellum (RF). C. Protein lysates from treated TH17 trophozoites were subjected to western blotting using anti-α-tubulin (α-Tub) or anti-GAPDH antibodies. D. CFSE-prelabelled trophozoites were co-cultured with *h*VECs (moi 1:3) for 30 min and the number of trophozoites bound per 1,000 *h*VECs was calculated as shown in the bar graph (mean ± SD). All assays were repeated three times. Significant differences were statistically analyzed by the Student’s t-tests with *P*< 0.01(**), *P*<0.05 (*), and ns, not significant.

Before the trophozoite transforms into the amoeboid form, it explores and anchors to *h*VECs via its axostyle, which is an important structure participating in locomotion and cell division [43]. To study whether the axostyle anchoring is involved in cytoadherence, the parasite was treated with 10 μM of tubulin polymerization inhibitor (TPI) to block the microtubule assembly in the axostyle. According to the IFA of DMSO control parasite, α-tubulin was localized in the axostyle of TH17 with minor signals in the flagella (Fig 6A). In the parasite treated with TPI, the α-tubulin distribution in the axostyle was localized to numerous punctate signals in the cytoplasm with parts around the nucleus (Fig 6A), whereas it did not affect immunostaining for the α-actin cytoskeleton. As shown by SEM, the parasite anchoring on *h*VECs via the axostyle was observed in the DMSO-treated specimen, whereas the parasite was rounder with no visible axostyle extending beyond the cell body in the TPI-treated TH17 trophozoites (Fig 6B). Meanwhile, the real-time imaging showed that the behavior of axostyle anchoring on *h*VECs was abrogated by TPI (S8 Video), and its behavior like T1 isolate (S7 Video). Western blot analysis revealed that the TPI challenge did not affect the overall α-tubulin expression in the TH17 trophozoites (Fig 6C) but inhibited *h*VECs binding by 40% in a 30-min cytoadherence assay (Fig 6D). So axostyle anchoring may have a role in the initial stage of cytoadherence to *h*VECs. Notably, the T1 trophozoites with intact axostyles were still less anchored on and barely adhere to *h*VECs (Fig 2B), implying that axostyle anchoring might be a transition step essential for initiating but insufficient for promoting *T*. *vaginalis* cytoadherence.

### Motility of *T*. *vaginalis*

**Fig 7:**
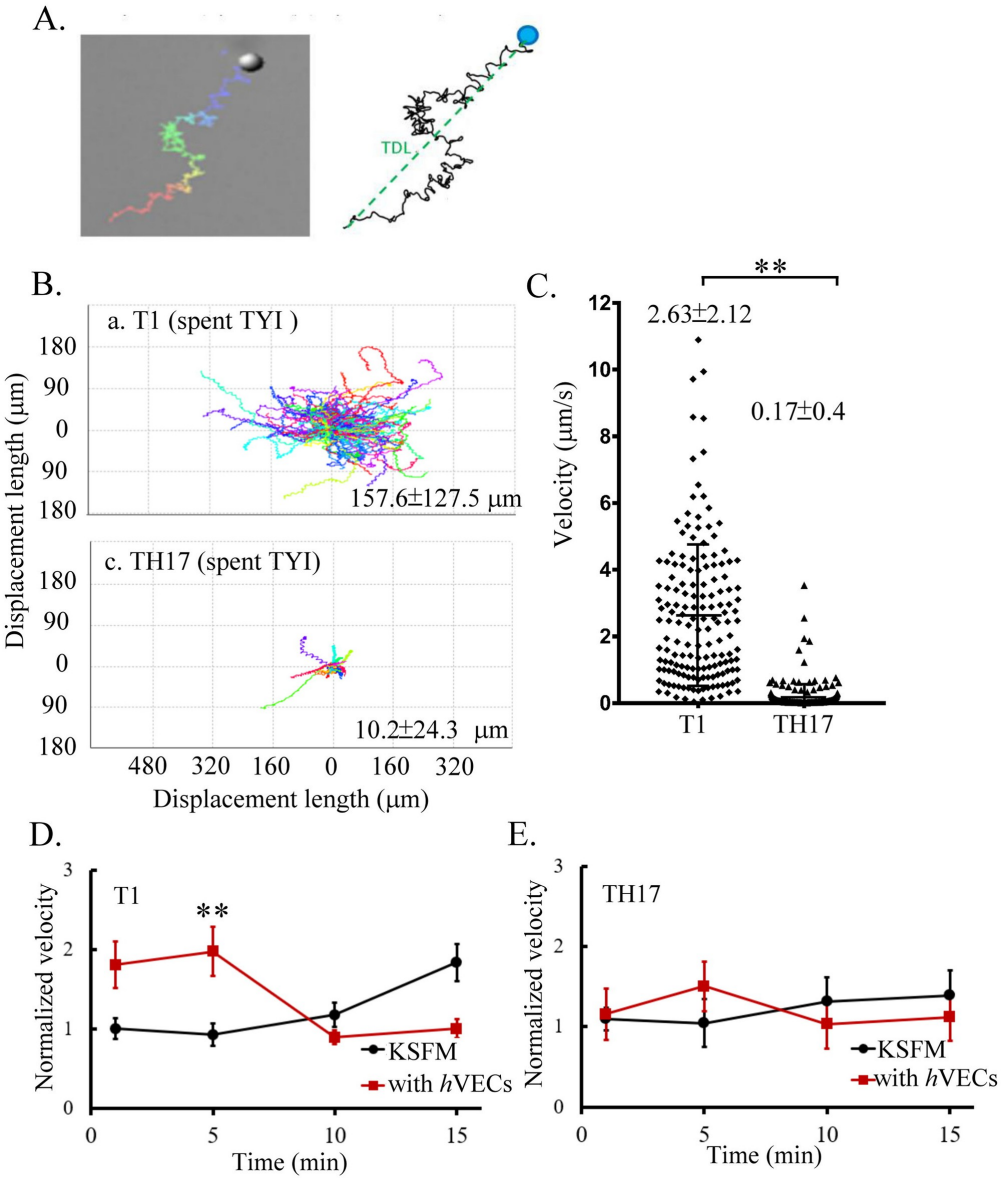
Motility of nonadherent versus adherent *T*. *vaginalis* isolates. The tract of a single TH17 or T1 trophozoite migrating on a glass slide for 1 min is depicted in A. The migratory paths from ~50 trophozoites are shown in B, with the average track displacement length (TDL). The average velocities with standard deviations were statistically analyzed by Student’s t-tests as shown in C. The relative velocity of T1 in D. or TH17 in E., the trophozoites cultured in KSFM or with *h*VECs over 15 min was normalized by the average velocity of those in 1 min post-incubation in KSFM. The assays were repeated three times. For D. and E., the statistical analysis was measured by Bonferroni post hoc tests (n = 10 to 30 for each group). The error bars represent standard deviations, *P*<0.01(**) and *P*<0.05(*).

Except for the amoeboid migration specifically observed in the adherent isolate, axonemal motility is a common motile movement adopted by adherent and nonadherent isolates (S1 and S2 Videos). The axonemal motility of individual trophozoites was monitored for 1 min to determine the characteristic track displacement length (TDL) and velocity of a specific isolate (Fig 7A). The motility of individual T1 trophozoites is diverse, with a TDL ranging between ~30 to 300 μm, and a velocity ranging between 0.5 to 4 μm sec^-1^, with an average of ~2.63 μm sec^-1^. Most TH17 trophozoites moved rather slowly, with an average velocity of ~0.17 μm sec^-1^ and TDL within ~35 μm (Fig 7B and 7C), indicating that the adherent isolate migrated more slowly than the nonadherent isolate. The parasite motility may inversely correlate with its ability to anchor on *h*VECs via the axostyle, and this needs to be considered in the study of host-parasite interactions. T1 trophozoite motility transiently speeded up at the initial 5 min post-infection with a velocity 2-fold faster than the KSFM control, then slowed down to the initial speed. By contrast, T1 trophozoite motility gradually sped up until 15 min in the KSFM control free of *h*VECs (Fig 7D), suggesting the parasites speed up in the presence of *h*VECs. A similar tendency was observed in TH17 motility but with less change than T1, suggesting that the motility of the adherent isolate may be less responsive to the *h*VECs than the nonadherent isolate (Fig 7E).

### Epigenetic divergency in adherent versus nonadherent *T*. *vaginalis* isolates

**Fig 8:**
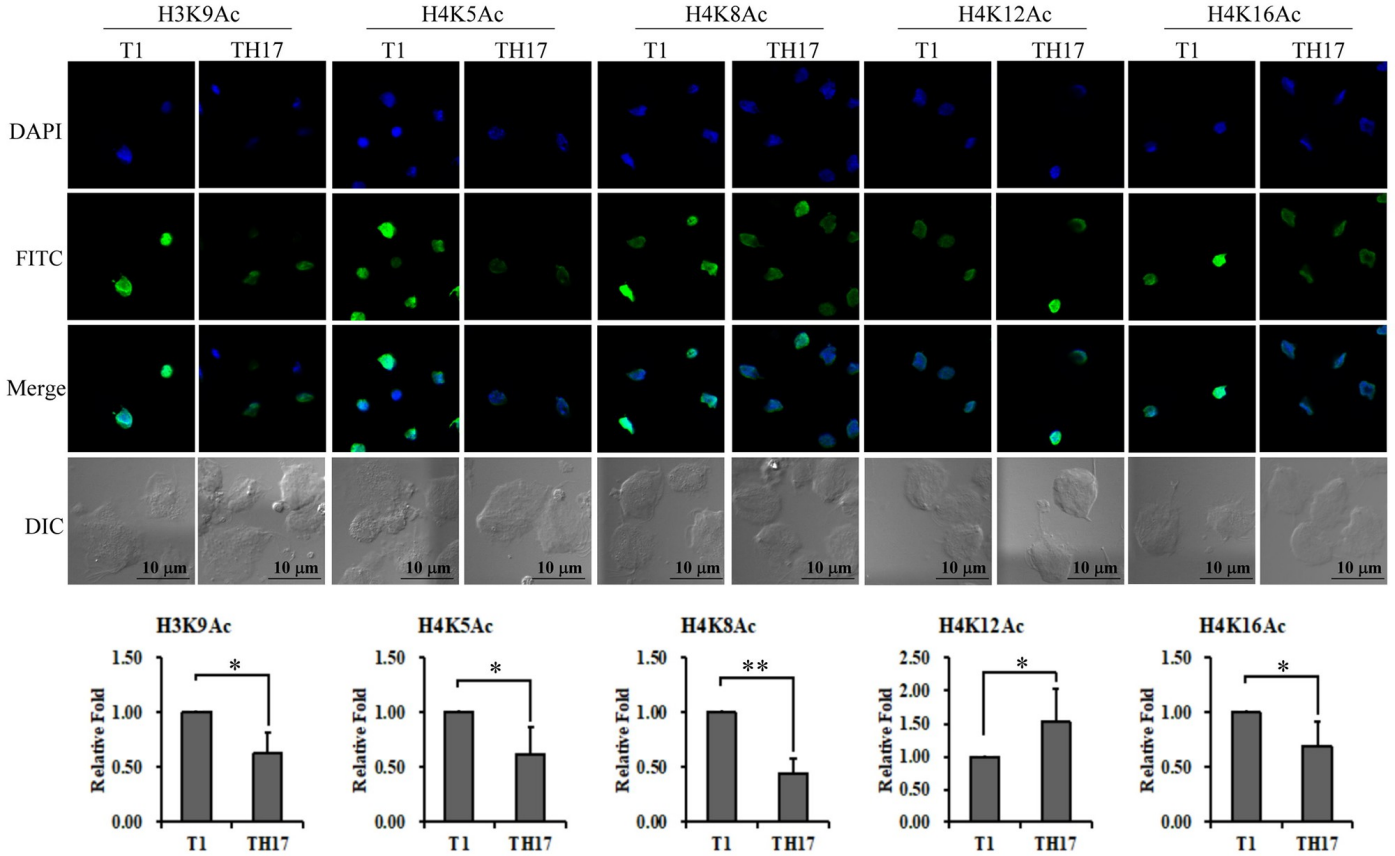
Epigenetic divergence of adherent versus nonadherent *T*. *vaginalis* isolates. Site-specific histone acetylation in *T*. *vaginalis* was examined by IFA using the antibodies as indicated on the top of each panel. The cell morphology was observed by microscopy at DIC mode. Scale bar represents 5 μm. The relative intensities of nuclear signals were quantified as shown in the bar graphs (n = 3, mean ± SD). Significant differences were statistically analyzed by Student’s t-test with *P*< 0.01(**) or *P*< 0.05 (*).

Regarding epigenetic regulation in *T*. *vaginalis*, the overall acetylation of K9, K14, K18, K23, and K27 on histone H3 has been previously reported to regulate the transcription of *BAP1* and *BAP2* adhesion protein genes in *T*. *vaginalis* B7268 strain [20]. To test whether the two isolates in our study may exhibit variation in histone acetylation at other potential sites, the site-specific histone acetylation at H3K9, H4K5, H4K8, H4K12, or H4K16 in the parasite was individually determined by IFA (Fig 8). Around 2 to 5-fold variation in signal intensity was detected among individual trophozoites, with an average intensity of acetylated H3K9, H4K5, H4K8, or H4K16 substantially higher in T1 than TH17 trophozoites. By contrast, that of acetylated H4K12 was significantly higher in TH17 than in T1 trophozoites. Given histone acetylation is associated with the cell cycle, the signal intensity of histone acetylation was heterogeneous in some individuals. The single-site acetylation may not be appropriate to differentiate the unsynchronized nonadherent and adherent isolates under our test condition.

### Mycoplasma symbiosis detection

**Fig 9:**
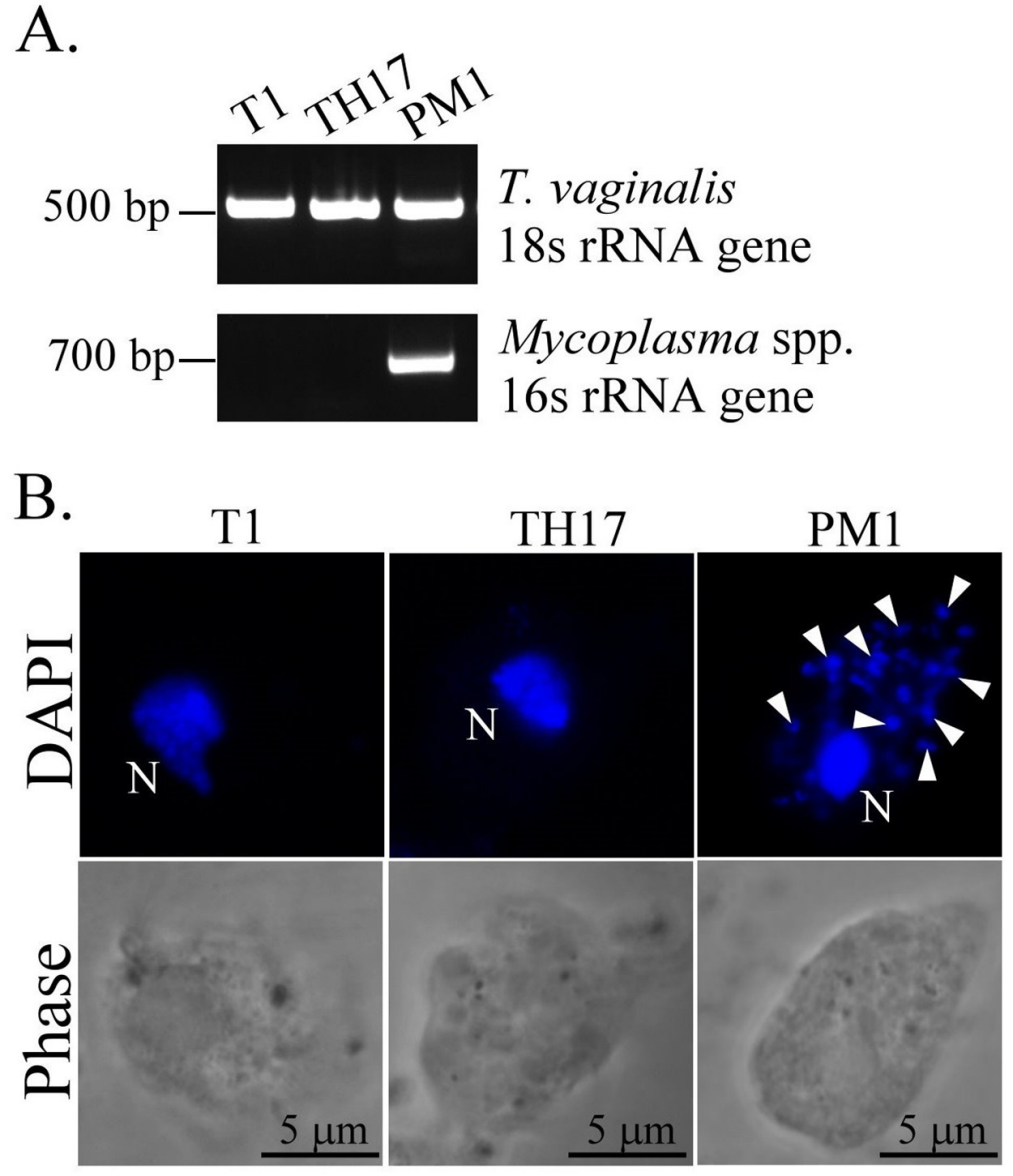
Mycoplasma symbiosis detection in the TH17 isolate. A. DNA extracted from T1, TH17, and PM1 isolates were amplified by PCR using specific primers for *T*. *vaginalis 18s rRNA* or *Mycoplasma* spp. *16s rRNA* genes. The PCR products were separated in a 1% agarose gel. B. The trophozoites from T1, TH17, and PM1 isolates were fixed on a glass slide and stained with DAPI for confocal microscopy. The cell morphology was visualized by phase-contrast microscopy. The *T*. *vaginalis* nuclei are indicated by the letter N, and the Mycoplasma DNA puncta are indicated by white arrowheads. Scale bar represents 5 μm.

A recent study reported that transcription of BspA-like proteins, surface proteins, and exosome proteins in *T*. *vaginalis* was modulated by Mycoplasma symbiosis [44], which implicates parasite pathogenicity. Therefore, we examined whether TH17 isolate contains Mycoplasma, showing that the Mycoplasma 16s rRNA gene was only amplified in the Mycoplasma-positive PM1 strain (Fig 9A) and Mycoplasma-like DNA puncta were only detected in the PM1 cytoplasm (Fig 9B), indicating that the TH17 isolate has no Mycoplasma symbiosis.

**Fig 10:**
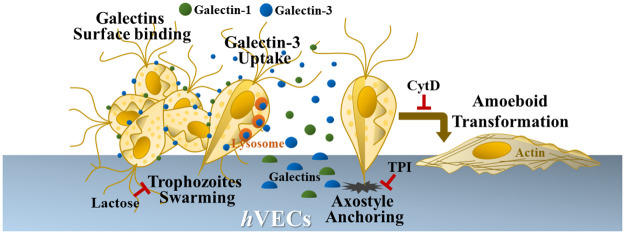
The differential host-parasite interaction of *T*. *vaginalis*. On infection, *T*. *vaginalis* may trigger the surface expression and secretion of galectins from *h*VECs. The extracellular galectins may initially bind on the parasite surface, then galectin-3 is internalized and enclosed in the lysosomes inside the trophozoites of adherent isolate. The extracellular galectins bound to the parasite might trigger the aggregation of trophozoites on *h*VECs, as this is diminished in the presence of lactose. In the adherent isolate, the predominant flagellate-amoeboid transition, as well as cytoadherence, were simultaneously suppressed by CytD, speculating that actin cytoskeleton-based behavior may mediate cytoadherence. When axostyle microtubule assembly was disturbed by TPI, the axostyle anchoring was abolished with the reduced cytoadherence, suggesting that axostyle anchoring might be an early event required for cytoadherence. For *T*. *vaginalis*, it is speculated that the cytoskeleton and cell surface adhesion molecules may coordinate their cytoadherence, so these factors should be considered in the study of host-parasite interactions and provide new insights into the host colonization of *T*. *vaginalis*.

In summary, our data show that *T*. *vaginalis* induces the surface expression and secretion of galectins from the host cells. The extracellular galectins initially bind *T*. *vaginalis* and might agglutinate parasites onto *h*VECs [25], but only the TH17 adherent isolate can internalize galectin-3 into the lysosome-like vesicles inside the parasite. The actin cytoskeleton organization is required for the cytoadherence of this parasite to *h*VECs, and anchoring behavior via axostyle may play a critical role critical in the initial cytoadherence (Fig 10). Upon infection, some extracellular factors may trigger the parasite to change motility. Differential histone acetylation reveals the subtle discrepancies in epigenetic background of these isolates. Also, the TH17 innate cytoadherence is not associated with Mycoplasma symbiosis. Whether these distinctive features coordinate colonization or the uropathogenic capacity of this parasite warrants further study.

## Discussion

Studies on *T*. *vaginalis* cytoadherence have mainly focused on the characterization of the ligand-receptor relationship between the parasite and host cells [45, 46]. Several putative adhesion molecules have not been identified in *T*. *vaginalis* [18–24]. By contrast, lipoglycans on *T*. *vaginalis* have been demonstrated to be the ligands binding to host galectin-1 or galectin-3 [25, 26], yet the surface expression of the galectins on host cells remains elusive. In addition, cytoadherence involves the action of a cadherin [22], a Rhomboid protease [23], a Legumain protease [18], exosomes [16], histone acetylation [20], and protein palmitoylation [47]. Together, these observations suggest that cytoadherence in *T*. *vaginalis* is more complicated than simply a ligand-receptor relationship. The transcription of multiple BspA and hypothetical surface proteins genes, as well as α-actin, α-actinin, and α-tubulin genes were instantly up-regulated over twofold in the parasite post-exposure to human cells, suggesting that *T*. *vaginalis* may coordinate adherence molecules with the cytoskeletal regulator to optimize cytoadherence upon contact with host cells [48]. It has been demonstrated that overexpression of BspA and Pmp domain-containing proteins in the nonadherent T1 isolate increases cytoadherence [21].

Typically, the binding activity of *T*. *vaginalis* trophozoites is evaluated based on the data collected from a single time point [18–21], and nonadherent or adherent isolates are used in gain- or loss-of-function assays, respectively, to infect the host cell lines but whether the time point selected to evaluate cytoadherence is optimal for a particular isolate and host cell is rarely addressed. In this study, trophozoite cytoadherence of adherent TH17 isolate was detected at 1 min post-infection and increased over 1 hr with around a 6-fold difference from the initial sampling point and over a 30-fold difference from the nonadherent T1 isolate (Fig 2). For nonadherent T1 isolate, cytoadherence over the infection course remained minimal with the levels fluctuating only 2–3 fold above the background. Thus, a 2-fold difference in the gain-of-function assays for nonadherent isolates may not be sufficient to support the contribution of particular factors in cytoadherence [19, 22].

The cytoadherence of adherent trophozoites to *h*VECs reported herein can be divided into an initial discrete phase followed by a swarming phase (Fig 2C) but only the second swarming phase was inhibited by lactose, not sucrose (Fig 2C), indicating that secreted galectins may be responsible for the parasite swarming [25]. As the epithelium cells in the urogenital tract, TH17 trophozoites also bind HeLa cells with a tendency similar to *h*VECs but less parasite aggregation (S1A Fig). Quantification of galectin-1 and galectin-3 secreted from *h*VECs and HeLa cells before and post-infection revealed that both galectins were secreted at much higher levels from *h*VECs than HeLa cells (S1B Fig), explaining why the swarming phase was less observed in HeLa cells on infection. The levels of secreted galectins decreased during the initial phase, returning to baseline levels or higher during the second phase of infection. Regarding cytotoxicity, there was only a slight change in LDH activity in the culture supernatant over the 60 min post-infection (S1C Fig), implying that the extracellular galectins do not leak from the damaged *h*VECs. Together, these data support that *T*. *vaginalis* interacts with different types of host cells to induce divergent reactions. Also, secreted galectins may be uptaken by *T*. *vaginalis* after binding on trophozoite surface as observed in Fig 3C and 3D. The uptake of galectin by the parasite may be the way associated with the observations that live parasites could deplete secreted galectin-3 [26]. Moreover, the capacity of individual trophozoites to bind recombinant galectins varied greatly (Fig 3F), indicating that the abundance of lipoglycans on individual trophozoites is heterogeneous and may not be relevant to cytoadherence. Notely, *T*. *vaginalis* infection rapidly induced the surface expression of galectin-1 and galectin-3 on *h*VECs, but not in close proximity to adherent trophozoites (Fig 3A), suggesting that the surface galectins may have a function other than the receptors for the cytoadherence. In human cells, galectin-1 and galectin-3 bind distinct glycoprotein receptors to induce T-cell and thymocyte death, of which CD45 and CD71 are involved in the galectin-3-induced T-cell death, and CD7 is required for the galectin-1-induced T-cell death [49]. Galectin-1 and galectin-3 lack the domains for transmembrane or GPI anchoring and they usually reside on the cell surface by binding surface glycoconjugates or proteins. In this study, the differential galectin-1 and galectin-3 distribution on *h*VECs surface is likely due to binding glycoconjugates differentially distributed on *h*VECs surface, which might activate *h*VECs in different ways.

Galectin is devoid of a signal sequence and its secretion is through a non-classical intracellular trafficking pathway that bypasses the Golgi complex [50]. *Trypanosoma cruzi* modulates the expression and secretion of galectin-1 leading to the inhibition of the proinflammatory mediator secretion by macrophages, thereby acting as a negative regulator to restrict host immunity on *T*. *cruzi* infection. *T*. *cruzi* infection also promotes galectin-3 expression with a high level of galectin-3 correlating with cardiac extracellular matrix remodeling leading to chronic cardiomyopathy [51].

*T*. *vaginalis* depleted extracellular galectins from the cervical epithelium cell culture in 24 hr [26]. In contrast, *T*. *vaginalis* induced the surface expression and secretion from host cells as soon as 15 min post-infection, possibly explaining the significant parasite swarming on *h*VECs post-30-min-infection (Fig 3B). Furthermore, galectin-3 uptake by the parasite occurred within 30 min, and the galectin was enclosed in lysosome-like vesicles (Fig 3E), the acidic organelles containing numerous proteolytic enzymes responsible for protein degradation [52]. The phagocytosis of yeast by *T*. *vaginalis* was reported to occur in lysosomes through the mediation of lectin-like receptors [53]. Thus, galectin-3 in *T*. *vaginalis* lysosomes might also be digested. The reduced galectin-3 may decrease the proinflammatory chemokine IL-8 production and thus diminish the recruitment of neutrophils or macrophages to the inflammation site for the clearance of extracellular protozoan pathogens, supporting the previously proposed mechanism [26]. Recently, the secretion of several *T*. *vaginalis* cysteine proteases was identified to be via an unconventional secretory pathway through lysosomes [34]. However, more investigations are needed to determine whether lysosomal degradation is adopted by *T*. *vaginalis* to evade the host immune system.

Flagellate-amoeboid transformation in the TH17 parasite occurs on contact with a glass slide or host cells (S2 Video). Except for the amoeboid trophozoites lying flat on host cells, the rounded-up flagellates anchored on host cells via the axostyle might be an important step for host-parasite interactions (Fig 5). However, the T1 trophozoite with intact axostyle still manifested behaviors such as less-anchoring and nonadherence to *h*VECs, suggesting that some other factors like actin-mediated amoeboid morphogenesis may physically increase the contact area and attachment strength, optimizing *T*. *vaginalis* cytoadherence activity [54]. Meanwhile, the anchored parasite seems to interact with nearby *h*VECs or trophozoites via membrane protrusions and flagella (Fig 4). Except for *h*VECs, various types of epithelium cells interact with *T*. *vaginalis*, including HeLa [46], MDCK [55], and Caco-2 [56] cells. Also, *T*. *vaginalis* can phagocytose yeast [53], lactobacilli, erythrocytes, and leukocytes [57]. Again, we speculate that *T*. *vaginalis* may interact with different types of host cells but trigger various downstream reactions.

Before the amoeboid transformation, the parasite explores and anchors to *h*VECs via its axostyle, an important structure for flagellate locomotion and cell division [43], whereas the adherent isolate moves slower, suggesting that the motility of the parasite isolate inversely correlates with its ability to anchor to *h*VECs via the axostyle. Motility is probably one of the major determinants to distinguish the adherent activity of *T*. *vaginalis*, so these factors should be considered in the study of host-parasite interactions. For a typical adherent trophozoite, the axostyle constantly touches the glass surface and it moves much slower only traversing within a much smaller region than a nonadherent counterpart (S5 Video).

Unlike the TH17 isolate, the motility of T1 trophozoites is more rapid after coculture with *h*VECs, implying that secreted factors from host cells may accelerate parasite migration, thus increasing the contact frequency in a nonadherent isolate to promote infection. In *Leishmania mexicana*, the flagellar motility of infective metacyclic promastigote is more active than its non-infective procyclic promastigote, and their flagellar swimming direction and speed are modulated by macrophage-derived stimuli with chemotaxis towards host immune cells [58]. To date, none of the structural components constituting the motor in the flagella of *T*. *vaginalis* have been elucidated, so whether the motility of the four anterior and one recurrent flagellum are regulated by a similar mechanism is unclear. The axostyle in *T*. *vaginalis* has been proposed to be involved in cell division [43]. Apart from cilia and flagella, the motility of certain flagellates may exploit repetitive expansion and contraction of microtubules in the axostyle to propagate undulatory waves for locomotion [59]. In high eukaryotes, expression and post-translational modifications of α-tubulin control microtubule assembly responsible for cell movement [60, 61].

In contrast to an earlier report on the potential regulation of cytoadherence by histone acetylation [20], we found that histone acetylation at a single site is unlikely to determine cytoadherence since the level of site-specific acetylation is heterogeneous among individuals. Since the T1 isolate was employed in both studies, the discrepancy in observed histone acetylation is likely due to the more dilute antibody used in our study or different antibody characteristics from the other report [20]. Nonetheless, histone acetylation may not serve as an appropriate marker for differentiating cytoadherence of *T*. *vaginalis*. In addition, Mycoplasma symbiosis was recently reported to correlate with the pathogenicity of *T*. *vaginalis* [44] and may increase parasite virulence. However, our results suggest that the symbiont is not the determinant conferring TH17 intrinsic cytoadherence. What underlies the cytoadherence in different *T*. *vaginalis* isolates remains an intriguing question, so whether the distinctive features reported herein play roles in the pathogenicity or virulence of this parasite remains to be further investigated.

## Supporting information

S1 Video Axonemal motility of the T1 isolate. The motility of T1 trophozoites in a T25 flask was dynamically recorded by real-time imaging with the capture rate at 1 frame per 30 sec over 1hr.
(MP4)

S2 Video Amoeboid migration of the TH17 isolate. TH17 trophozoites were cultured in a T25 flask for 1 hr before analysis and the amoeboid migration was recorded by real-time imaging with a capture rate of 1 frame per 30 sec over 10 min.
(MP4)

S3 Video Uptake of galectin-3 by *T*. *vaginalis*. TH17 trophozoites were pre-incubated with FITC-conjugated recombinant galectin-3 and then co-cultured with *h*VECs. The intracellular galectin-3 condensed to multiple vesicle-like compartments in *T*. *vaginalis* were visualized by real-time imaging.
(MP4)

S4 Video Observations of T1 migration by real-time imaging. The migration of T1 trophozoites on a glass slide was imaged by a time-lapse microscope.
(MP4)

S5 Video Observations of TH17 migration by real-time imaging. TH17 trophozoite migration on a glass slide was imaged by a time-lapse microscope.
(MP4)

S6 Video The observation of the host-parasite interaction of the TH17 isolate by real-time imaging. TH17 trophozoites co-cultured with *h*VECs were imaged by a time-lapse microscope, showing the long-lasting anchor of the parasite to *h*VECs via the axostyle.
(MP4)

S7 Video The observation of the host-parasite interaction of the T1 isolate by real-time imaging. T1 trophozoites co-cultured with *h*VECs were imaged by a time-lapse microscope.
(MP4)

S8 Video The observation of the host-parasite interaction of TH17 trophozoites treated with TPI. TH17 trophozoites treated with TPI were co-cultured with *h*VECs and the parasite behavior was observed by real-time imaging at 1 frame per 30 sec over 1 hr.
(MP4)

S1 Fig Host-parasite interaction of *T*. *vaginalis* with HeLa cells. A. CFSE-labeled TH17 trophozoites were co-cultured with *h*VECs and HeLa cells at a moi of 1:3 for the cytoadherence assay. The samples were fixed at specific time points for fluorescence microscopic observation. The number of bound trophozoites and clustered paraistes foci (circled by white-dashed line) per 1,000 *h*VECs were measured as shown in the bar graphs. B. The secreted galectin-1 and galectin-3 in the conditioned medium supernatant collected from *h*VECs and HeLa cells co-incubated with TH17 trophozoites at the indicated time intervals were detected by ELISA. C. The supernatants from *h*VECs or HeLa cells co-cultured with TH17 trophozoites were collected for the LDH cytotoxicity assay and the supernatants from host cells treated with Lysis Solution were used as the positive control (100%), and relative host cell cytotoxicity versus positive control at different time points post-infection were measured as shown in the bar graph. The data are presented as the mean ± SD. All assays were repeated three times. Diffepprences were statistically analyzed by Student’s t-test, with *P*<0.01(**) and *P*<0.05(*).
(TIF)

S1 Data Raw data Western blot and gel. This PowerPoint file includes raw images of western blotting (Figs 4C and 6C) and agarose gel (Fig 9A).
(PPTX)

S2 Data Statistic data. This Excel file includes the values measured for statistical analysis in Figs 2A, 2B, 2C, 3B, 4B, 4C, 4D, 6C, 6D, 7B, 7C, 7D, 7E, 8 and S1A–S1C.
(XLSX)
