# Supplementary figures and images for: Distinct features of the host-parasite interactions between nonadherent and adherent Trichomonas vaginalis isolates
Source: PLoS Negl Trop Dis. 2023 Jan 3;17(1):e0011016. doi: 10.1371/journal.pntd.0011016 (PMC9810166; doi:10.1371/journal.pntd.0011016)

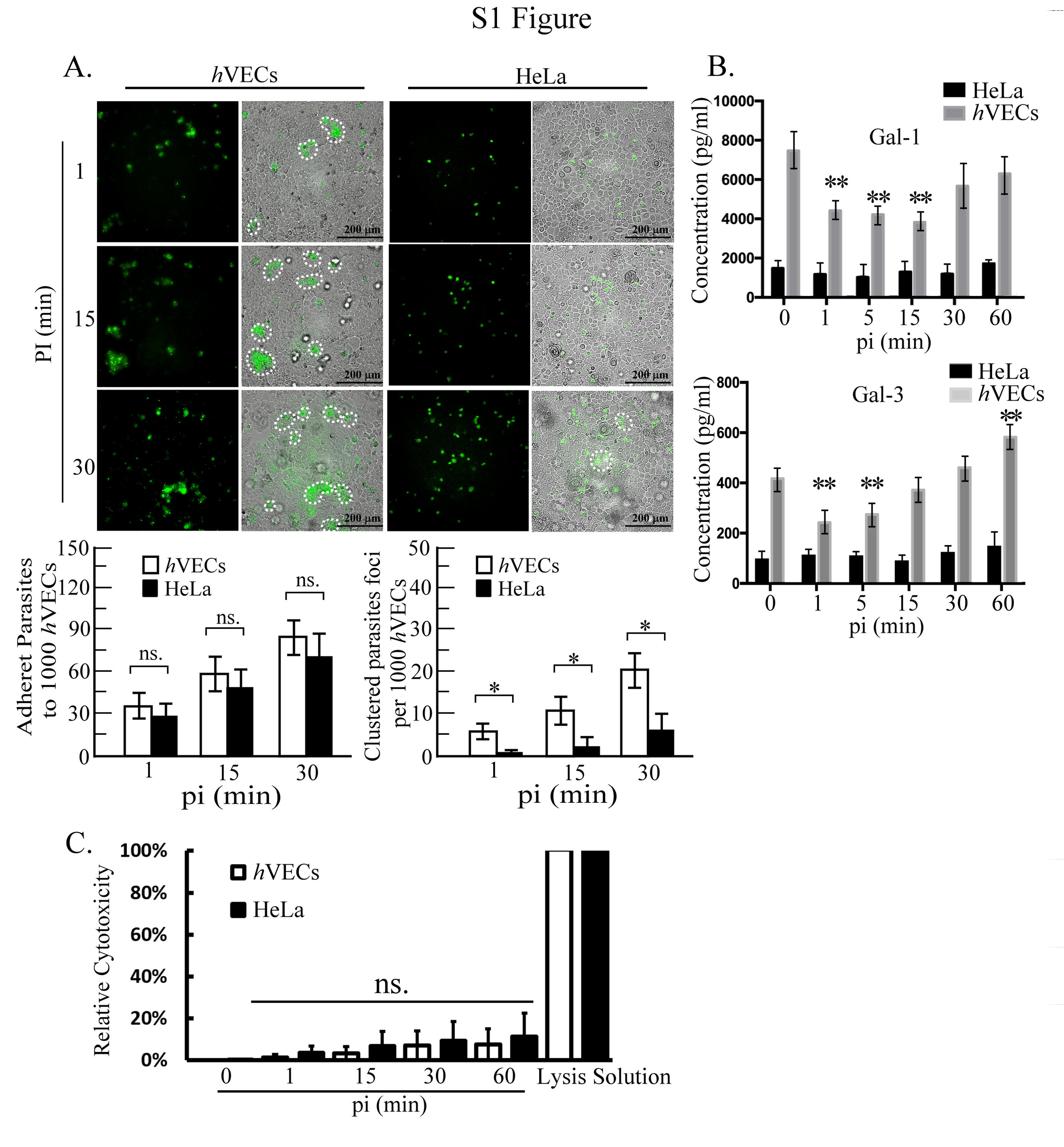

Supplement: S1 Fig — A. CFSE-labeled TH17 trophozoites were co-cultured with hVECs and HeLa cells at a moi of 1:3 for the cytoadherence assay. The samples were fixed at specific time points for fluorescence microscopic observation. The number of bound trophozoites and clustered paraistes foci (circled by white-dashed line) per 1,000 hVECs were measured as shown in the bar graphs. B. The secreted galectin-1 and galectin-3 in the conditioned medium supernatant collected from hVECs and HeLa cells co-incubated with TH17 trophozoites at the indicated time intervals were detected by ELISA. C. The supernatants from hVECs or HeLa cells co-cultured with TH17 trophozoites were collected for the LDH cytotoxicity assay and the supernatants from host cells treated with Lysis Solution were used as the positive control (100%), and relative host cell cytotoxicity versus positive control at different time points post-infection were measured as shown in the bar graph. The data are presented as the mean ± SD. All assays were repeated three times. Diffepprences were statistically analyzed by Student’s t-test, with P<0.01(**) and P<0.05(*). (TIF) [file pntd.0011016.s009.tif]
