# Supplementary material for: Distinct features of the host-parasite interactions between nonadherent and adherent Trichomonas vaginalis isolates
Source: PLoS Negl Trop Dis. 2023 Jan 3;17(1):e0011016. doi: 10.1371/journal.pntd.0011016 (PMC9810166; doi:10.1371/journal.pntd.0011016)
Supplement: S1 Data — This PowerPoint file includes raw images of western blotting (Figs 4C and 6C) and agarose gel (Fig 9A). (PPTX) [file pntd.0011016.s010.pptx]

## Slide 1
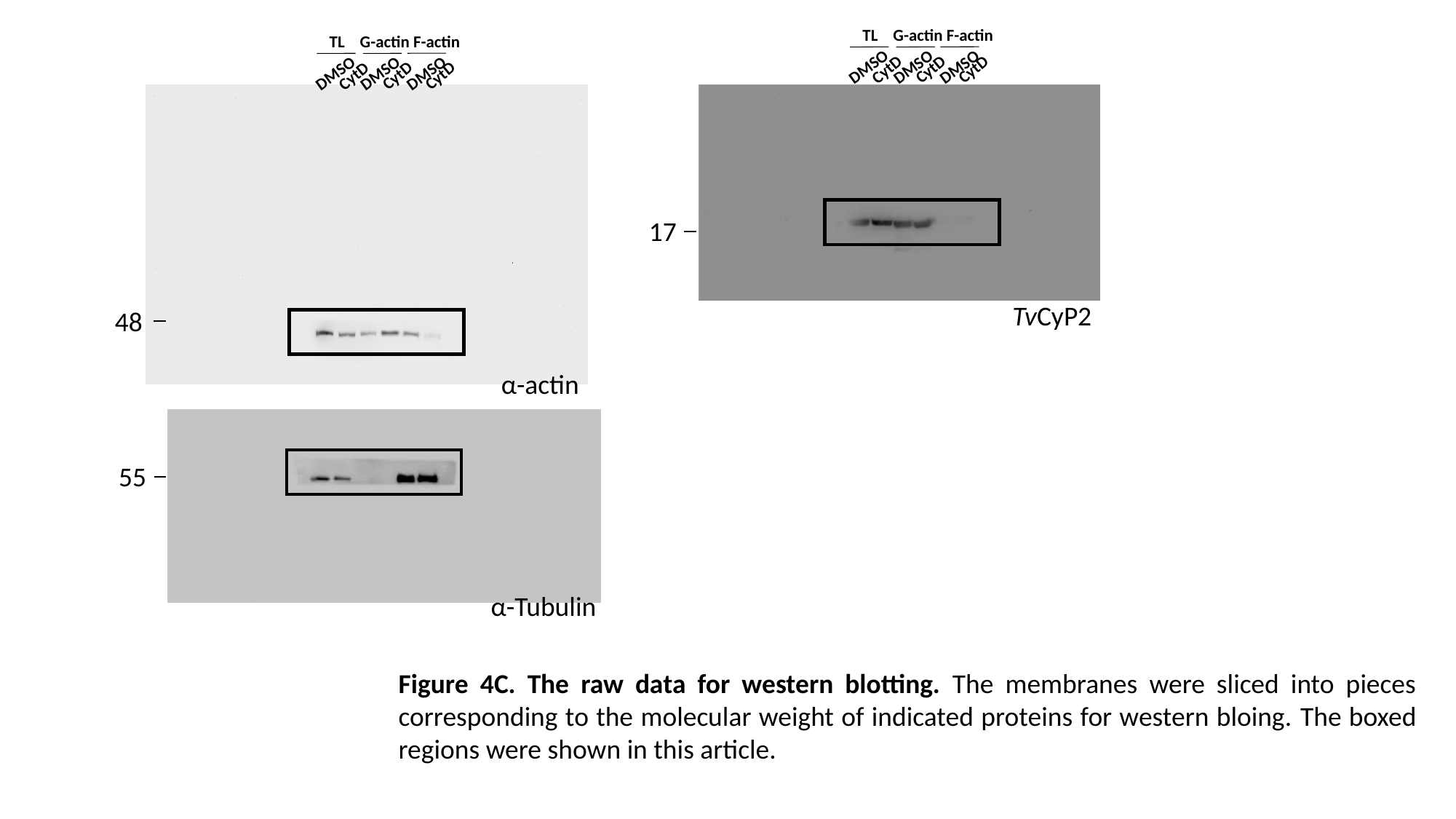

TL G-actin F-actin
DMSO
DMSO
DMSO
CytD
CytD
CytD
TL G-actin F-actin
DMSO
DMSO
DMSO
CytD
CytD
CytD
17
TvCyP2
48
α-actin
55
α-Tubulin
Figure 4C. The raw data for western blotting. The membranes were sliced into pieces corresponding to the molecular weight of indicated proteins for western bloing. The boxed regions were shown in this article.

## Slide 2
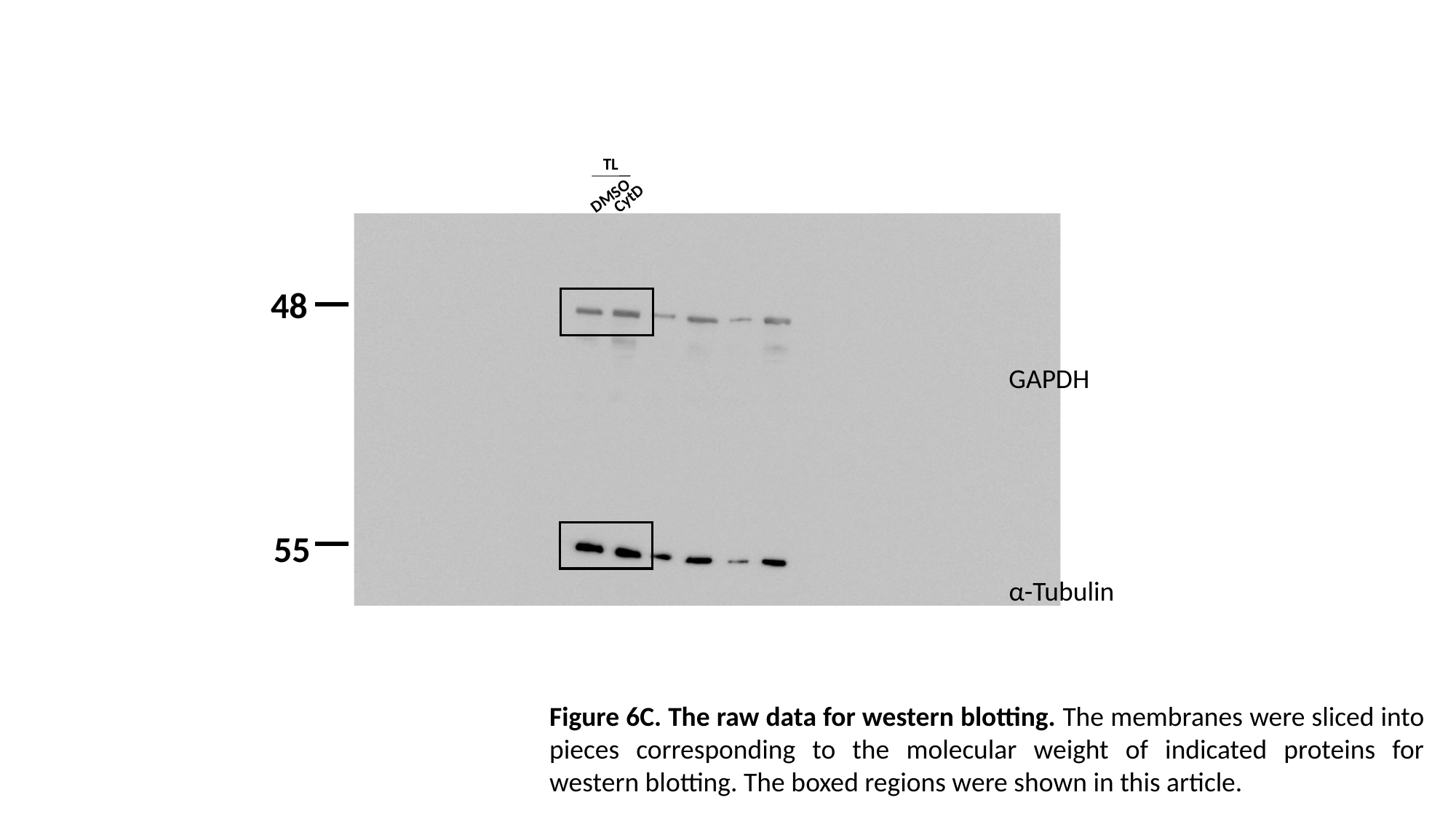

TL
DMSO
CytD
48
GAPDH
55
α-Tubulin
Figure 6C. The raw data for western blotting. The membranes were sliced into pieces corresponding to the molecular weight of indicated proteins for western blotting. The boxed regions were shown in this article.

## Slide 3
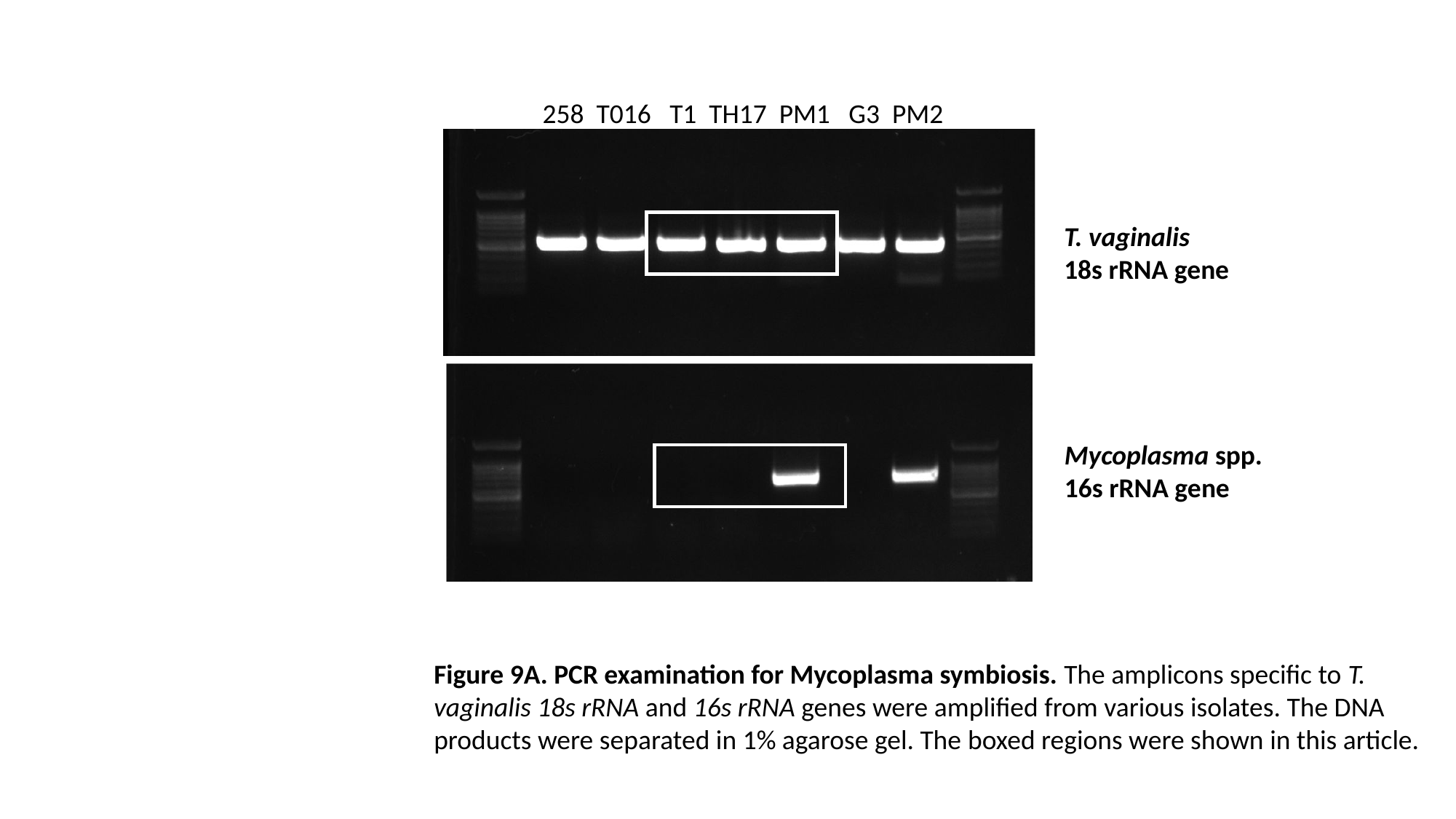

258 T016 T1 TH17 PM1 G3 PM2
T. vaginalis
18s rRNA gene
Mycoplasma spp.
16s rRNA gene
Figure 9A. PCR examination for Mycoplasma symbiosis. The amplicons specific to T. vaginalis 18s rRNA and 16s rRNA genes were amplified from various isolates. The DNA products were separated in 1% agarose gel. The boxed regions were shown in this article.
